# Effects of stochasticity and division of labor in toxin production on two-strain bacterial competition in *Escherichia coli*

**DOI:** 10.1371/journal.pbio.2001457

**Published:** 2017-05-01

**Authors:** Benedikt von Bronk, Sophia Anna Schaffer, Alexandra Götz, Madeleine Opitz

**Affiliations:** Center for NanoScience, Faculty of Physics, Ludwig-Maximilians-Universität München, München, Germany; The Hebrew University of Jerusalem, Israel

## Abstract

In phenotypically heterogeneous microbial populations, the decision to adopt one or another phenotype is often stochastically regulated. However, how this stochasticity affects interactions between competing microbes in mixed communities is difficult to assess. One example of such an interaction system is the competition of an *Escherichia coli* strain C, which performs division of labor between reproducers and self-sacrificing toxin producers, with a toxin-sensitive strain S. The decision between reproduction or toxin production within a single C cell is inherently stochastic. Here, combining experimental and theoretical approaches, we demonstrate that this stochasticity in the initial phase of colony formation is the crucial determinant for the competition outcome. In the initial phase (t < 12h), stochasticity influences the formation of viable C clusters at the colony edge. In the subsequent phase, the effective fitness differences (set primarily by the degree of division of labor in the C strain population) dictate the deterministic population dynamics and consequently competition outcome. In particular, we observe that competitive success of the C strain is only found if (i) a C edge cluster has formed at the end of the initial competition phase and (ii) the beneficial and detrimental effects of toxin production are balanced, which is the case at intermediate toxin producer fractions. Our findings highlight the importance of stochastic processes during the initial phase of colony formation, which might be highly relevant for other microbial community interactions in which the random choice between phenotypes can have long-lasting consequences for community fate.

## Author summary

Competition is the dominant interaction type between species of bacteria. Bacterial toxin-mediated competition is often accompanied by a division of labor between toxin-producing cells and reproducers within a species. In populations with large cell numbers, the stochastic dynamics that determine if an individual cell switches into the toxin-producing state are often unnoticed. Consequently, we know little about how stochastic effects influence bacterial competition. Here, combining experimental and theoretical efforts, we study the competition of a toxin-producing strain with a toxin-sensitive strain. By correlating the initial conditions—at near single-cell level—to the macroscopic competition outcome, we investigate both the importance of the division of labor as well as the influence of the stochastic toxin production dynamics on competition outcome. Our results highlight the impact of the initial phase of competition as a major determinant for the success of the toxin-producing population.

## Introduction

Interactions like cooperation and competition between different organisms govern ecosystem dynamics, influencing ecosystem composition [1], maintenance of biodiversity [1–4], and the microbiota–host relationship [5–9]. Despite the detailed knowledge of individual interaction mechanisms on the one hand [10,11] and large-scale sequencing based microbiome studies on the other hand [7–9,12,13], the fundamental problem in microbial ecology is the need for predictive model systems that combine experiments with theoretical modelling to explain how ecosystem dynamics emerge from interactions between single cells [14,15]. Simple bacterial model systems allow the elimination of distorting factors and enable the investigation of fundamental processes governing these dynamics, such as stochasticity, under well-defined experimental conditions [2–4,16,17].

In principle, cooperative [18,19] and competitive [10,20] interactions can occur between members of the same or of different species [21] and are mediated by various mechanisms [11,14,21–24]. Cooperative interactions are often realized by phenotypic heterogeneity, which is a division of labor within isogenic populations [24], in which only a subpopulation produces a public good. Competitive interactions are achieved indirectly by competition for resources such as nutrients and space [22] but can also act directly by production of bacteriocins [7]—protein-based toxins produced by many microbes [10].

One class of bacteriocins, the colicins, are synthesized only by a subpopulation of the producer strain [25–27]. The ColicinE2 is one of these heterogeneously produced colicins by *E*. *coli*. Here, similar to many other colicins [25], phenotypic heterogeneity is a consequence of the regulation via the noisy SOS response [26,28,29], which can be triggered by the inducing agent mitomycin C (MitC) [25], with higher MitC levels increasing the fraction of toxin producers [30]. The dynamics of ColicinE2 production have been investigated in detail [30]. Individual bacteria switch into the toxin-producing state stochastically [31]. Once a cell has switched into the producing state, it produces the toxin for approximately 60 min and subsequently releases the produced toxin into the environment upon cell lysis [30,32]. Cells that do not produce the toxin continue to reproduce. Hence, two different types of cells (toxin-producing and reproducing cells) are present, and a division of labor is established. Especially at small cell numbers, the stochastic switching dynamics can have important consequences. However, for large cell numbers, the stochastic nature of toxin production is less important, and the system approaches a steady state with constant producer fraction.

The combined action of the two cell types (toxin-producing and reproducing cells) can be seen as a specific type of indirect cooperation that does not rely on communication but in which the tuning of producer fractions is determined by the architecture of the gene regulatory network and its response to an external signal (MitC concentration) [30]. Hence, the production of ColicinE2 by *E*. *coli* incorporates both direct competition (by production of toxins) and cooperation (by means of division of labor/phenotypic heterogeneity).

The competition between colicinogenic and susceptible strains of bacteria has been studied as a model system for allelopathy [33–35]. Theoretical studies highlighted the importance of colicin production cost, toxin effectiveness, and initial strain ratios as determinants of the outcome of competition [33]. Furthermore, long-term coexistence of both strains was found to emerge only in structured habitats [33]. More recent studies included experiments and focused on coexistence and biodiversity of toxin producer, toxin-sensitive, and/or resistant strains [1,3–5, 36]. However, these studies mainly neglected the cooperative aspect of toxin production by the ColicinE2 producer strain (C strain). In particular, to our knowledge, the influence of heterogeneity and stochasticity in toxin production on competition outcome and C strain success is largely unexplored, and quantitative experimental validation of theoretical predictions remains lacking.

**Fig 1:**
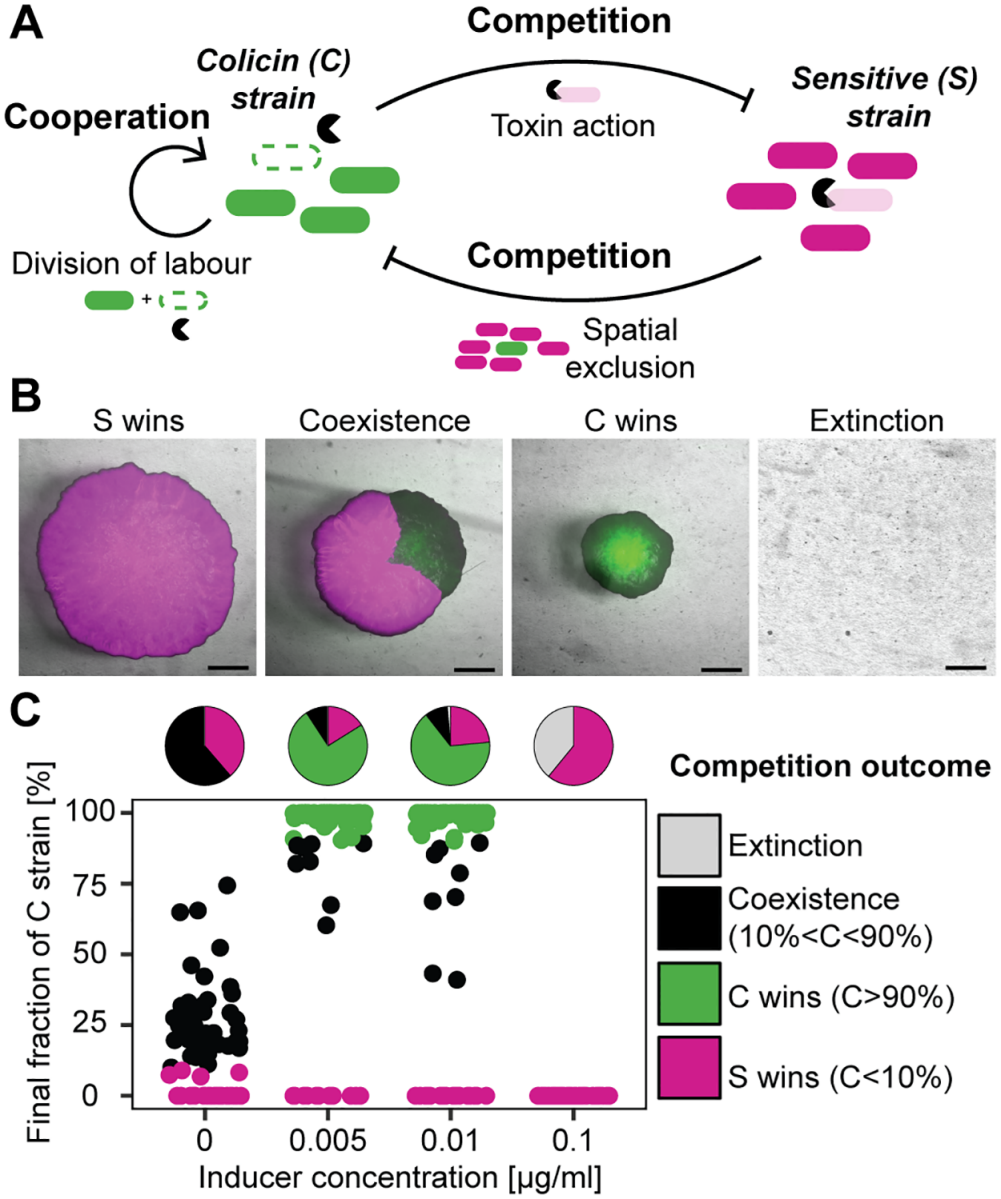
Two-strain competition, strategies, and outcome. C–S competition results in four competition outcomes. The competition outcome distribution changes with increasing inducer concentration. (A) Interaction between a toxin-producing strain (C strain) and a sensitive strain (S strain) is characterized by inter-strain competition (spatial exclusion and toxin action) and intra-strain cooperation within the C strain (division of labor between reproducers and toxin producers). (B) Possible outcomes of competition experiments between sensitive (magenta) and producer (green) cells after 48 h (scale bar = 1 mm). Mitomycin C (MitC) concentrations from left to right: 0.01/0.0/0.01/0.01 μg/ml). (C) Final fraction of C after competition (dot plot) and classified outcomes (pie plots) demonstrate a transition from coexistence and dominance of S (0.0 μg/ml MitC) to dominance of the producer strain (intermediate inducer concentrations, 0.005 and 0.01 μg/ml MitC) and failure of the toxin production strategy (high inducer concentration, 0.1 μg/ml MitC).

To address this problem, we employed an experimental approach using a stereoscopic microscope with zoom functionality, which enables investigations over multiple length-scales. In particular, this setup allowed us to correlate well-defined initial conditions with the macroscopic competition outcome of bacterial range expansions, hence, studying the impact of stochastic effects during initial colony formation on competition outcome. Computational modelling of the interaction by means of stochastic, spatially extended, lattice-based simulations supplemented the experiments. Using these two tools, we investigated the interaction of a C strain with a strain sensitive to the toxin (S strain) (Fig 1A). This system exhibits both indirect intra-strain cooperation between reproducers and toxin producers within the C strain population and inter-strain competition between the C and S strain, mediated via toxin action and denial of access to resources by spatial exclusion.

Our data revealed that the competition dynamics can be divided into two phases. In the initial phase of competition, at small cell numbers, stochastic effects originating from the stochastic toxin production dynamics and random initial positioning influenced the number of viable C clusters at the colony edge. In the second, deterministic phase, the degree of division of labor and the number of viable C clusters at the colony edge determined the final outcome of competitions.

## Results

Mixed bacterial communities of fluorescently labelled C and S strains were prepared on solid growth media with an initial C:S ratio of 1:100. This ratio boosted the competitiveness of the S strain by facilitating spatial exclusion. At the same time, this ratio allowed us to investigate if and how the C strain is able to outcompete the S strain, in case the S strain is initially dominant in cell number. We imaged the communities using a stereoscopic microscope with a zoom function, which enabled us to acquire time-lapse recordings of the competition from the near single-cell level up to mature, macroscopic colonies. We then extracted colony area and colony composition using customized image and data analysis software (S1 Fig, Methods).

To assess the influence of cooperation within the C strain described above, as well as stochasticity in toxin production on competition outcome and C strain success, we performed range expansion experiments at four concentrations of the inducing agent MitC (0.0, 0.005, 0.01, 0.1 μg/ml). After 48 h of competition, we observed four qualitatively distinct outcomes based on the relative area occupied by the particular strain: domination by C or S, coexistence, and extinction of both strains (Fig 1B, S2 Fig and S1–S4 Videos). Domination signifies that one strain occupies over 90% of the colony area, coexistence denotes occupancies of between 10% and 90%, and occupation of a total area of less than 10^6^ μm^2^ constitutes extinction. This was in contrast to competitions performed under well-mixed conditions (S3 Fig), in which toxin release by the C strain lead to a growth arrest of the S strain in all cases.

In our experiments, we observed a strong dependence of competition outcome distribution on the inducer concentration (Fig 1C). Without MitC, either S won, or the competition resulted in coexistence. At low inducer concentrations, C won in the majority of cases, while at high inducer concentrations C succumbed, and one observed either domination by S or extinction of both strains (Fig 1C). Hence, domination by C was only observed at intermediate inducer concentrations. Although we could identify clear differences between the outcome distributions, we unexpectedly observed multistability—the presence of multiple competition outcomes under similar initial conditions (the same inducer concentration).

**Fig 2:**
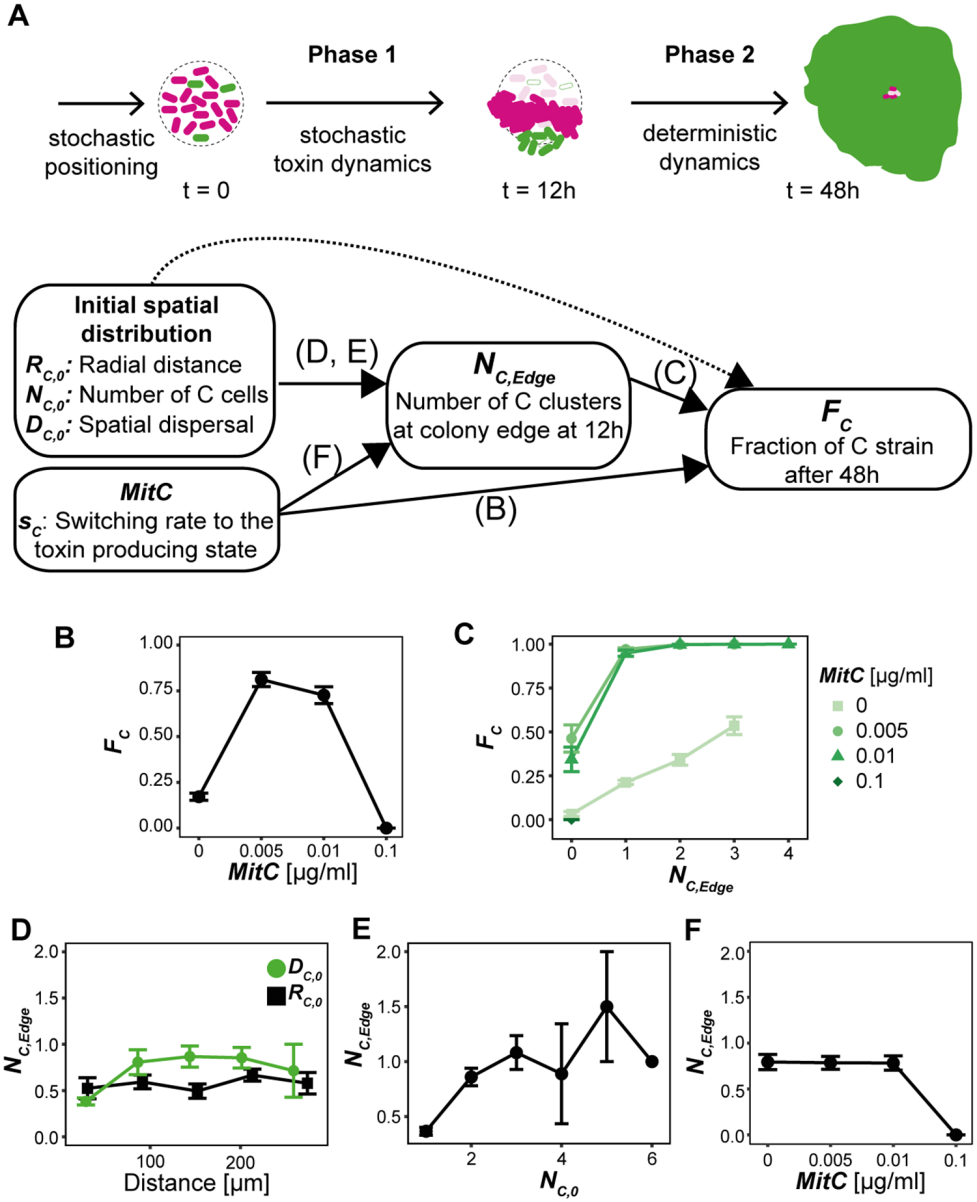
Two-phase analysis of competition outcome determinants in experiments. (A) The interaction dynamics are divided into two phases. In phase one, the stochastic switching from replication to toxin production of the ColicinE2 producer strain (C strain) determines the number of viable C clusters at the edge of the colony after 12 h, *N*_*C*,*Edge*_. In phase two, the interaction follows deterministic dynamics in which *N*_*C*,*Edge*_ and *MitC* are suitable predictors for *F*_*C*_, the fraction of the C strain after 48 h, whereas the spatial initial conditions only weakly influence *F*_*C*_. (B) For intermediate mitomycin C (MitC) concentrations (as a proxy for toxin producer fraction), the average *F*_*C*_ is significantly increased. (C) The *N*_*C*,*Edge*_—*F*_*C*_ diagram grouped and colored according to MitC concentrations. (D) Dependence of *N*_*C*,*Edge*_ on the average distance of C cells from the colony center *R*_*C*,0_ (black squares) and the average distance of C cells from the C cell center *D*_*C*,0_ (green circles) as proxys for initial spatial conditions. Data was binned into five equal-sized bins. (E) The relation between initial C cell number *N*_*C*,0_ and *N*_*C*,*Edge*_. (F) The effect of *MitC* concentration on average *N*_*C*,*Edge*_. (Data points show the average value, error bars denote standard error). E/F data represent the average over all four MitC concentrations.

**Fig 3:**
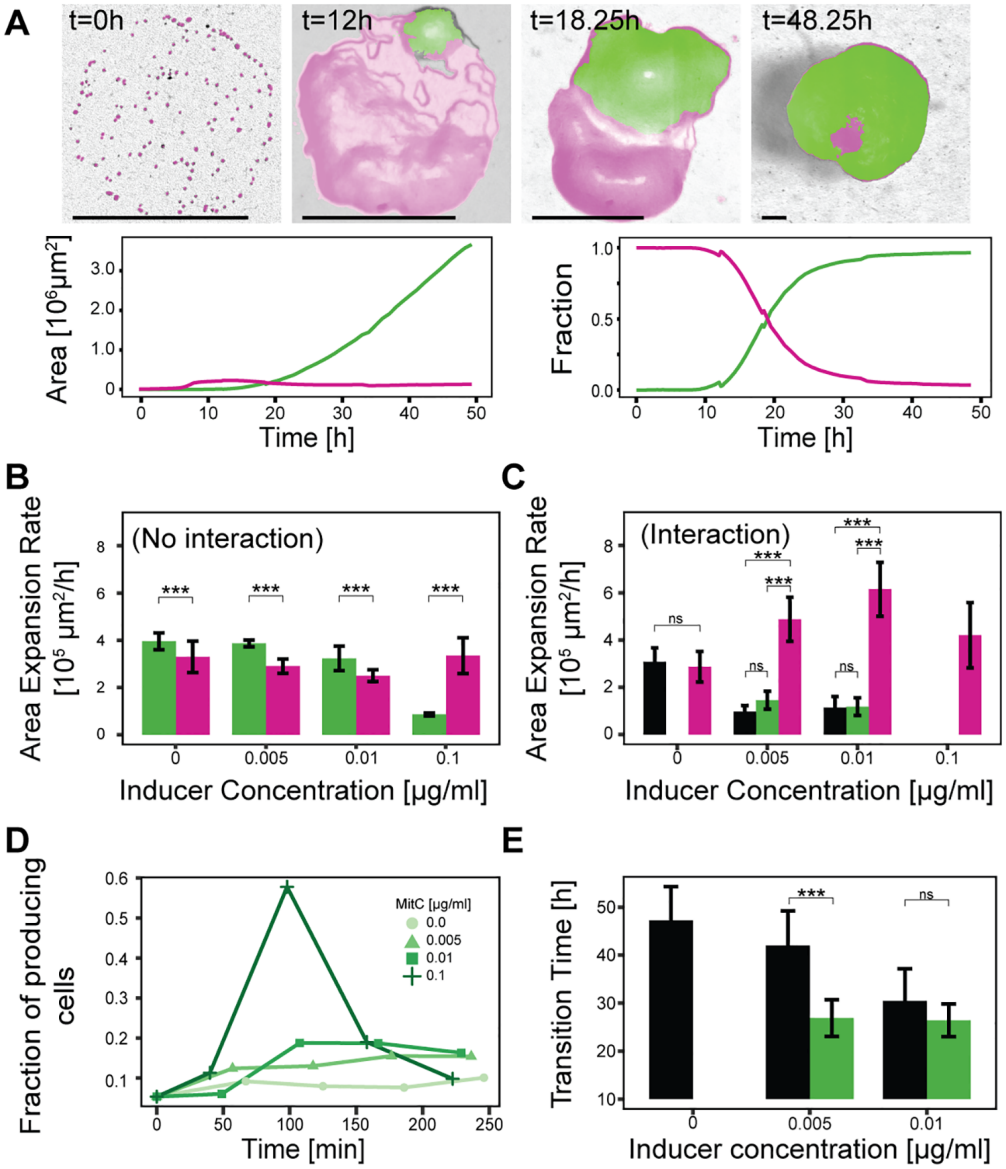
Characterization of interaction dynamics. The increase of the toxin producer fraction and not the ColicinE2 producer strain (C strain) growth rate determines the change in outcome distributions. Unless otherwise stated, colors indicate competition outcome (magenta: sensitive strain, green: producer strain, grey/black: coexistence), error bars represent mean ± standard deviation. (A) Sample image time series and corresponding area and relative fraction curves show C domination outcome (0.01 μg/ml MitC). Scale bar represents 400 μm. (B) Average expansion rates of single-strain colonies obtained in control experiments (no interaction). (C) Effective expansion rates of the entire S–C colony during competition (interaction present). Colors indicate the outcome of the competition: e.g., magenta indicates that >90% of S are present and responsible for colony expansion. (D) Temporal evolution of the producer fraction in the C population in high-resolution control experiments (Methods). (E), The dependence of transition time (time taken for C to capture more than 50% on the colonized area) on inducer concentration. (B, C, E: asterisks indicate significant differences as obtained by pairwise *t*-tests; not significant (ns): *p* > 0.05, ***: *p* < 0.001, for details see S1 Data).

**Fig 4:**
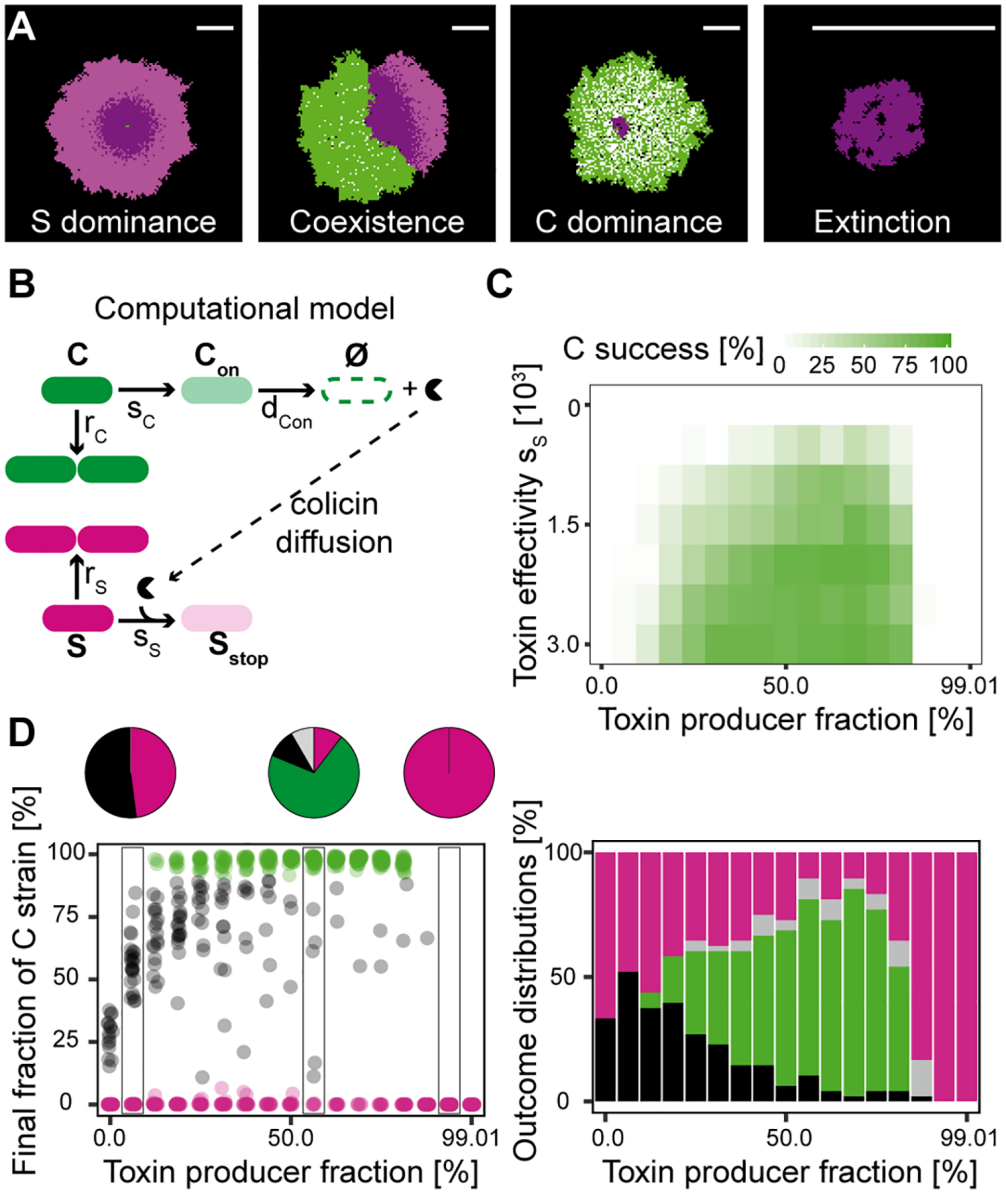
Theoretical analysis emphasizes the importance of intermediate toxin producer fractions for ColicinE2 producer strain (C strain) success. (A) The four different competition outcomes in simulations (bright magenta: living S cells, dark magenta: growth-inhibited S cells, green: reproducing C cells, white: toxin-producing C cells). Scale bar = 1 mm. Values of the switching rate s_**C**_ from left to right: 0.001/0.02/0.001622/synchronicity scenario I. (B) The interaction scheme underlying the theoretical model (Methods). (C) C dominance phase diagrams for toxin effectivity **s**_**S**_ and toxin producer fraction. (D) The final fraction of the C strain (dot plot) and classified outcomes (pie and bar plots) after simulated competition at fixed **s**_**S**_ = 1,500 and in dependence on toxin producer fractions are in accordance with experimental results (Fig 1C). The color code represents competition outcome and is identical to that in Fig 1C.

To gain a mechanistic understanding of the interaction, we characterized the dynamics of the competition quantitatively. First, we analyzed the impact of stochastic effects in both toxin production and initial positioning on competition outcome (Fig 2). In a second step, we used experimental time-lapse data (Fig 3) as well as computational modelling (Fig 4) to investigate competition parameters that dictate the deterministic competition dynamics, such as growth rate, division of labor (toxin producer fraction) within the C strain, and toxin sensitivity. This enabled us to disentangle the influence of deterministic and stochastic effects and to identify the origin of multistability.

Stochastic effects that are mainly important in this C–S competition are the random initial spatial positioning of the C strain within the C–S colony and the stochastic switching from the replicating to toxin-producing phenotype of C cells at low cell numbers (Fig 2A). Our method enabled us to precisely determine the position and number of C cells in initial colonies and subsequently analyze their influence on competition outcome. Surprisingly, we found that the fraction of C cells in the colony after 48 h *F*_*C*_ is only weakly correlated to various initial spatial measures, such as initial C cell number *N*_*C*,0_, individual C cell distance to the colony center $\left|{\vec{x}}_{C,0,i}\right|$, average distance of C cells from the center *R*_*C*,0_, and the average distance of C cells from the center of all C cells *D*_*C*,0_ (Fig 2A dotted arrow, S4 Fig, Methods, and see S1 Data for correlation table).

In contrast, the second source of stochasticity, the stochastic toxin dynamics, was observed to strongly influence the final C fraction *F*_*C*_ (Fig 2B). For small cell numbers, stochastic switching has a big influence on the toxin production dynamics of individual C cells and can decide the fate of the single S–C communities. Qualitatively, this can be understood when considering different scenarios (S2 Fig): Early toxin production prevented C’s success in case there were too few C cells left to proliferate. Consequently, the S strain dominated or the whole S–C colony went extinct. In case of delayed toxin production, a considerable C strain population had formed, and enough reproducing C cells remained after toxin release to maintain the C strain population. This resulted in C domination or coexistence outcomes.

These observations inspired us to take a closer look at the initial phase of colony formation (phase one: t < = 12 h) where stochastic effects play an important role due to small cell numbers. We found that we could quantify our qualitative observations by *N*_*C*,*Edge*_, the number of viable C cell clusters at the colony edge after 12 h. Linear regression showed that the buildup of viable C clusters at the colony edge was significantly influenced by each of the spatial variables *N*_*C*,0_, *R*_*C*,0_, *D*_*C*,0_, and *MitC* as a proxy for the switching rate into the toxin-producing state (Fig 2D–2F, Methods, and S1 Data).

We found that variations in *N*_*C*,*Edge*_ together with the influence of the inducer concentration *MitC*, could explain the observed multistability (Fig 2C). As we will describe in the subsequent paragraphs, the deterministic dynamics were strongly influenced by the inducer concentration that determined the interaction regime. However, due to the stochastic nature of single-cell dynamics and initial spatial positioning, the formation of viable C cell clusters was subject to noise. Given a favorable deterministic regime (0.005, 0.01 μg/ml MitC), we can distinguish three cases: (1) *N*_*C*,*Edge*_ ≥ 1 lead to success of the C strain or transient coexistence, (2) *N*_*C*,*Edge*_ = 0 and there was no viable C cluster remaining lead to domination of the S strain, (3) *N*_*C*,*Edge*_ = 0 and there was no C cell cluster remaining and the random spatial distribution lead to unlikely but possible complete extinction. In cases of transient coexistence, growing C clusters were not large enough to cover over 90% of the area within the time-frame of our experiments but will take over in the long run. In the uninduced deterministic regime (0.0 μg/ml MitC), C success was prevented due to limited toxin action, and we only observed S success and coexistence with a linear dependence of *F*_*C*_ on *N*_*C*,*Edge*_ (Fig 2C). In the highly induced case (0.1 μg/ml MitC), buildup of viable C cell clusters was suppressed (Fig 2F), and depending on the spatial initial position of the S strain, S could survive, or we observed extinction.

To formally analyze the effects of the different variables, we employed a linear statistical model incorporating the variables *MitC*, *N*_*C*,*Edge*_, *N*_*C*,0_, *R*_*C*,0_ and *D*_*C*,0_ and their interactions to explain *F*_*C*_ (Methods, S1 Table). We found that significant effects stem from *MitC*, *N*_*C*,*Edge*_, their interaction, and the interaction term of *N*_*C*,*Edge*_ and *N*_*C*,0_. As expected from the previous results, large and medium effect sizes (based on *η*^2^ statistic) were only attributable to *MitC* and *N*_*C*,*Edge*_, respectively. In order to verify our findings with higher significance, we performed simulations (see below for details) with 16 random initial conditions *IC*, repeated 30 times each, over a range of 17 different switching rates *s*_*C*_. Intriguingly, we could observe different outcomes of the competitions under the very same initial conditions (*s*_*C*_ = 0.02, example in S4C Fig), underlining the result that the spatial initial conditions do not fully determine the competition outcome. Analogously to our experimental analysis, *N*_*C*,*Edge*_ was determined, and the response variable *F*_*C*_ was modelled in a linear statistical model (Methods, S1 Table). Here, all variables and their interaction terms are statistically significant, but only *s*_*C*_ and *N*_*C*,*Edge*_ have large *η*^2^ effect sizes (see S1 Table). This supports the experimental result that spatial initial conditions act only indirectly via *N*_*C*,*Edge*_ on the final C fraction *F*_*C*_.

In order to probe the relation between stochastic and deterministic phases, we performed experiments (at 0.005 μg/ml MitC) and simulations with increased initial density, as inoculation density is known to influence colony pattern formation [37]. Intuitively, one expects a decrease in multistability, with competition outcome distributions becoming increasingly deterministic. However, our observations indicate a counteracting mechanism for intermediate densities (S5 Fig). For 2- and 4-fold initial density, S success was decreased, but we also observed an increased fraction of coexistence outcome in experiments. These cases of coexistence were believed to be transient, and are most likely explained by an increased S area that established before the toxin stops S’s growth. We speculate that during the 48 h of competition, the C strain did not have enough time to catch up, and the final fraction was below 90%. If we increased the density further, S’s head start might not have been enough to act against the increased probability to have high *N*_*C*,*Edge*_ values, and we observed mostly C success with few coexistence events. In contrast to these ambiguous results for the outcome distributions, we found a decrease in variance of the C fraction dynamics averaged over the time-course of the experiment, with increasing density (OD 0.1: 0.2146, OD 0.2: 0.1630, OD 0.4: 0.1231, OD 0.8: 0.1013) indicating more reproducible dynamics with increasing initial cell density. Note that the simulation results differed slightly from the experimental observations. First, with increasing initial cell density, spotting of the C-S mixture resulted in an increased coffee stain effect in experiments. Hence, the density is not homogeneously increased as is the case in the simulations. Secondly, in simulations in which viable C clusters were surrounded by dead S cells, preventing spatial expansion and dominance of the C strain, the total colony size remained very small, and the outcome was therefore classified as extinct.

Up to now, we have learned that stochastic effects in initial positioning and toxin production dynamics influence the number of viable C cell clusters at the colony edge after 12 h (*N*_*C*,*Edge*_). Furthermore, we found that *N*_*C*,*Edge*_ together with the inducer concentration *MitC* determining the interaction dynamics of the second phase strongly predicts the outcome of competitions. However, an exact analysis of the influence of *MitC* on the deterministic dynamics in the second phase was missing. Therefore, we investigated competition parameters that dictate the deterministic competition using experimental time-lapse data and computational modelling.

In the first step, we evaluated the growth rates (area expansion rates) for both strains in the absence of the competing strain (Fig 3B, Methods). We found that the growth rates for S and the uninduced C strain were similar to those seen in previous studies [3], with C growing slightly faster on average. At high inducer levels, C’s growth rate falls considerably, owing to increased toxin release with concomitant lysis [30]. We speculate that the observed increased growth rate of the S strain can be attributed to selection for fast growing S cells at high MitC concentrations. We furthermore determined the growth rates of the entire colonies in competition experiments (Fig 3C). We found that in cases of final S strain dominance growth of the entire mixed colony is enhanced compared to the single S colony growth upon induction with MitC. Hence, competition may promote growth of S due to the selective effect of sublethal colicin concentrations (S3D Fig). In competition experiments that lead to final C strain dominance, the growth rate of the entire mixed colony is reduced compared to the single C strain colony growth rate. Here, although the C strain is dominating in the end, its growth is reduced by initial spatial exclusion by S, which restricts the territory available to the former.

The reduced effective growth of C in competition experiments emphasized that growth rate alone cannot explain its dominance at low inducer concentrations. Hence, we assessed the dependence of the second parameter, producer fraction within the C strain population, on MitC concentration using high-resolution microscopy (Fig 3D, Methods and S6 Fig). The dynamics of toxin production have been studied in detail for liquid environments using single-cell fluorescence time-lapse microscopy [30]. Here, the strongest population response is observed 75 min after induction, and it was found that the fraction of cells producing the toxin increases with the external stress level (MitC). While in the absence of external stress, only a basal producer fraction was found with few cells producing the toxin; low external stress resulted in a significant fraction of cells producing and releasing the toxin. In contrast, high MitC concentrations lead to a synchronized response of all cells [30]. We verified, that this correlation between the external stress level and the fraction of toxin-producing cells within the C strain population was also present for the experimental conditions used in this study (Fig 3D). In the absence of MitC, an intrinsically low producer fraction (7.0 ± 1.5%) was detected. At low MitC concentrations, the mean producer fraction increased to 14.5 ± 1.9% (0.005 and 0.01 μg/ml MitC combined), while the highest inducer concentration leads to a synchronized response (57.8 ± 3.2%) with a subsequent collective decrease in producer fraction due to cell lysis, in accordance with previous studies [30]. This suggested that the observed shift in competition outcome distribution with varying inducer concentration can be attributed to the change in toxin-producer fraction.

To verify that, indeed, increased toxin production was determining the interaction dynamics of the second phase, we repeated our competition experiments with a strain S_YFP_ that is genetically identical to the C strain but lacks the original colicin-producing pColE2-P9 plasmid and hence is not able to produce the toxin (Methods). Lysis in this strain is achieved through the additional lysis gene expression from a reporter plasmid encoding a yellow fluorescent protein (YFP) [30]. For this S_YFP_–S competition, we found mostly dominance of the S strain and coexistence of both strains at all inducer concentrations (S7A Fig). Similar to this all-OFF scenario (no cell able to produce the toxin), also a scenario with all C cells producing the toxin (all-ON) reduced C strain success (S7B Fig). At a very high MitC concentration of 0.4 μg/ml, all cells produced the toxin simultaneously [30] which lead to the total extinction of both the C and S strain in nearly all competition experiments. This showed that, indeed, the change in producer fraction, and consequently the toxin amount released, was causing the shift in competition outcome distributions, and demonstrated the importance of division of labor for C strain success. To characterize individual interaction dynamics, we extracted the transition time from the relative occupied area curves (Fig 3E). The transition time was defined as the time it takes for C to capture more than half of the colonized area. We observed that average transition times were shorter for the higher inducer concentration and that long-term C dominance was possible only if the transition occurred early. This again emphasized that population fate was mostly determined in the initial phases of colony formation.

To corroborate our experimental finding of toxin-producer fraction as the key parameter of the second phase’s interaction dynamics determining C strain success, we developed a theoretical model of the competition and simulated the system with parameters we obtained in our experiments. We used a stochastic lattice-based model that enabled us to explicitly incorporate the stochastic positioning and phenotypic heterogeneity of the C strain (Methods and Fig 4). Phenotypic heterogeneity in the model is a result of stochastic switching [31] from the reproducing (normal) state ***C*** to the toxin-producing state ***C***_***on***_. Because of cell lysis accompanying toxin release, producing cells can only decay and cannot switch back in the model. Our numerical simulations reproduced the four different outcomes observed in competition experiments (Fig 4A, S5–S8 Videos) and demonstrated that dominance of the C strain could be achieved by only varying the switching rate ***s***_***C***_, i.e., the propensity of a reproducer cell to switch to the producing state (Fig 4C). In particular, upon altering ***s***_***C***_, which corresponds to varying the inducer concentration, we observed the same qualitative changes in the final C strain fraction and competition outcome as were seen in experiments (Fig 4D) for a constant toxin effectivity ***s***_***S***_. With low and high producer fractions, we observed no domination of the C strain, whereas the C strain took over for intermediate inducer concentrations. More explicitly, at ~50% toxin-producing C cells, we found mostly dominance of the C strain (Fig 4C) but also dominance of the S strain and coexistence of both strains. The question arose if one observed the same competition outcome if the C strain was able to release the toxin while growing, which is the case for other toxin-producing species [25]. In such a scenario, we found that the C strain dominates in nearly all cases (S7C Fig). Hence, the C strain is similarly successful if the toxin can be released without cell lysis by all toxin producers, even if the growth rate and the toxin sensitivity is reduced. This indicates that cell lysis bears a high cost for the toxin producer.

The third important interaction parameter, the toxin effectivity ***s***_***S***_, represents the total toxin impact and incorporates toxin amount released by the C strain and toxin sensitivity of the susceptible strain S. Because of its complexity, it is hard to exactly determine this parameter experimentally. However, its effect on the dynamics can be explored in the model. We simulated the system for a range of ***s***_***S***_ and ***s***_***C***_ values, yielding phase diagrams for each of the four competition outcomes (Fig 4C, S8A Fig). In agreement with our experimental findings, dominance of C was indeed prominent in regions of intermediate producer fractions. Furthermore, this held for a broad range of toxin effectivities ***s***_***S***_. Conversely, S dominance was most likely where C’s toxin production strategy failed. Coexistence was mostly found at low toxin producer fractions or low toxin effectivity. Extinction events occurred all over regions of the phase diagram where C prevailed and became more prominent for higher ***s***_***C***_ values. However, the simple computational model could not reproduce the high incidence of extinction events seen in experiments at high inducer levels. A more detailed model incorporating the synchronicity of the toxin-production response to external stress yielded higher numbers of extinction events (S8B Fig and S9 and S10 Videos), highlighting the fact that population extinction at high producer fractions requires a synchronous response.

Taken together, the theoretical model clearly showed that only varying the switching rate and thereby the toxin producer fraction within the C strain population is sufficient to explain why the C strain can be dominant at intermediate inducer concentrations (= intermediate toxin producer fractions).

**Fig 5:**
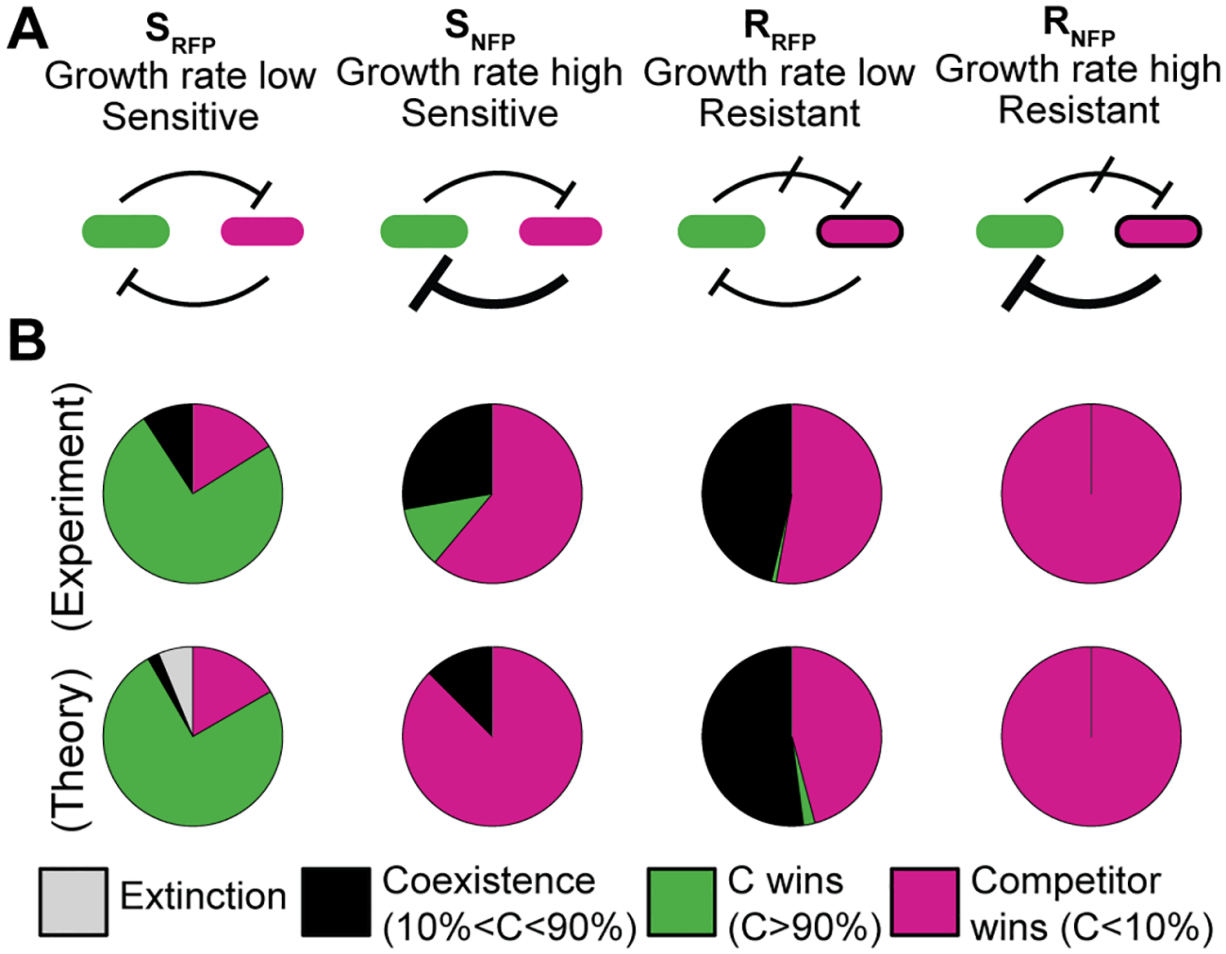
ColicinE2 producer strain (C strain) dominance depends on the competitor’s strategy. Experiments were performed at 0.005 μg/ml mitomycin C (MitC). Toxin resistance or induced growth enables the competitor to outcompete the C strain. Altering the background fluorescent marker expressed from fluorescent reporter plasmids in the respective strain, allows one to vary the growth rate of this strain. The expression of the red fluorescent protein (RFP) mCherry results in a significant growth rate reduction [3]. Strains indicated by no fluorescing protein (NFP) do not express a fluorescent protein. (A) Alternative competition strategies involving disablement of toxin action (R_RFP_, R_NFP_) and/or increased spatial exclusion (S_NFP_, R_NFP_) compared to the original toxin-sensitive strain (S strain) (S_RFP_). (B) Experimental (top) and simulated competitions (bottom) yield similar outcome distributions.

To explicitly test the influence of relative strain growth rate and toxin sensitivity, we repeated the competition of C with three other strains experimentally (at 0.005 μg/ml MitC) and varied the growth rate and toxin sensitivity parameters in simulations (Fig 5 and S9C Fig). Experimentally, this included altering the growth rate of the competitor and its sensitivity to the toxin (Methods). Boosting the growth rate of S (S_NFP_) increased the effect of its competition strategy, spatial exclusion, and improved the S strain’s competitiveness considerably. Resistance to the toxin (R_RFP_) neutralized the deleterious effect of C on its competitor and prevented dominance of C. Furthermore, increasing the resistant strain’s growth rate (R_NFP_) enabled it to consistently prevail over C. These results (Fig 5B) were confirmed by our theoretical simulations, using the appropriate growth rates and toxin sensitivities for the competitor mutants obtained in experiments (S9A Fig and S2 Table). This underlined the ability of our model to accurately predict competition outcome distributions.

Exploring the dependence of outcome probability on relative growth rate and toxin sensitivity further (S9C Fig), we found a trade-off between both parameters. The phase diagrams show a dividing line of coexistence along a diagonal axis that represents the growth rate and sensitivity trade-off. For instance, if a strain was sensitive and slow, it succumbed to the toxin producer. Conversely, a strain that was less sensitive did not need a high relative growth rate to thrive in competition. These insights can be used to predict the influence of growth rate changing fluorophore expression (S9A Fig) on the deterministic competition dynamics. The costly expression of red fluorescent protein (RFP) compared to no fluorescing protein (NFP) (S_RFP_ compared to S_NFP_) [38] influenced competition outcome considerably (Fig 5, S9A Fig and S2 Table) in accordance to our theoretical results. Likewise, we expect the relatively small decrease in growth rate of the C strain compared to the corresponding wild-type strain due to fluorophore expression (Methods) to only weakly influence the interaction.

Taken together, the results show that stochastic and deterministic effects can be disentangled. In the first phase (t < 12 h), at low densities, stochastic effects of toxin production and spatial distribution influence the number of viable C cell clusters at the colony edge after 12 h (*N*_*C*,*Edge*_). Together with a measure for the division of labor (MitC in experiments, *s*_*C*_ in simulations) the deterministic dynamics of the second phase can be well predicted. In this second competition phase, the strains simply expand in range afterwards, following deterministic dynamics governed by the effective fitness differences between the two strains [17].

## Discussion

Our results clearly showed, how stochastic processes influenced the initial phase of colony formation and how effective fitness differences stemming from division of labor within the toxin-producing population dictated the deterministic interaction dynamics of the second phase.

We demonstrated that the outcome of competitions fought under the same condition is not fixed [33–35]. Instead, we could observe four different competition outcomes in our experiments. This multistability was a result of the stochastic toxin-production dynamics. This can be understood if we disentangle the dynamics into two distinct phases. In phase one, the stochastic toxin-production dynamics of individual cells at early time points determine the fate of a given S–C colony by affecting the number of viable C cell clusters at the colony edge *N*_*C*,*Edge*_ (phase one, Fig 2). In the second phase, the stochastic toxin production manifests itself in a constant toxin-producer fraction constituting the division of labor/phenotypic heterogeneity. The exact degree of heterogeneity sets effective fitness differences between both strains and, together with the number of C clusters at the colony edge, it dictates the deterministic macro dynamics and thereby competition outcome distributions (phase two, Fig 2).

As a result, in case of sufficient toxin action (0.005 and 0.01 μg/ml MitC), the effective fitness differences were so large that once a viable toxin-producer cluster had formed at the colony edge (*N*_*C*,*Edge*_ > 0), it took over the colony in the long run. Hence, the coexistence observed at 48 h was only transient, S never won as we observed *N*_*C*,*Edge*_ > 0, and the dynamics were fully determined by the initial phase.

However, in the case of limited toxin action (0.0 μg/ml MitC), the effective fitness difference was not as clear. The observed coexistence was persistent as the effective fitness differences were not large enough to let the C strain take over during the time-course of our experiments (S2 Video) and after prolonged competition (>48 h). Furthermore, in the absence of MitC, we observed few cases in which the S strain won, although we had observed *N*_*C*,*Edge*_ > 0. These cases were in accordance to previous studies that showed how inevitable random fluctuations due to genetic drift can cut off access to the exterior for similarly expanding colony domains [39]. Therefore, we conclude that for the uninduced case, the initial phase does not fully determine the outcome but predicts the final fraction of the C strain well (see also Fig 2C).

By taking advantage of the multiscale functionality of the presented experimental method, we could investigate both the parameters for the deterministic dynamics as well as the influence of stochasticity in toxin production and initial conditions. In addition, we are confident that this method can be used to investigate a variety of interaction types in detail, thereby advancing our fundamental understanding of bacterial interactions.

The computational model used in this study nicely complemented our experiments. When used with experimentally observed parameters, it was able to predict competition outcome distributions in good accordance with the experimental observations. Variations of toxin-producer fraction, growth rate, and the switch from sensitive to resistant strains showed outcome distributions similar to experiments. Furthermore, the model could be exploited to investigate conditions that we could not create experimentally, such as varying toxin effectivity *s*_*S*_, replication of simulations with the very same initial conditions, and simulations of competitions with a non-lysing C strain.

Our conclusions about stochasticity as the origin of multistability are robust with respect to a wide range of different parameters influencing the deterministic interaction dynamics, such as toxin effectivity, growth rate, or exact switching rate. Obviously, the initial influence of stochasticity decreases with increasing density.

The observed trade-off between the beneficial effect of antibiotic production and the effective fitness disadvantage the producer strain exerts on itself is in accordance with Geradin et al. [36]. In this study, the fitness disadvantage was further increased by the presence of resistant bacteria.

In summary, our results show that stochasticity in the initial phase of colony formation can be the crucial factor that determines the ensuing population dynamics and consequently controls the final composition of the community. This is in accordance with a recent study of the effects of stochastic assembly on host-associated microbial communities [40] and underlines how random events at the single-cell level can influence the fate of microbial communities in the long run. Besides toxin production, there are many phenotypically heterogeneous traits in which decisions between one or another phenotype happen stochastically. Therefore, our findings are relevant for a broad range of microbial communities, in which the random choice between phenotypes during the initial phases of colony formation effectively fixes the community's composition and ultimate fate.

## Methods

### Bacterial culture

The *E*. *coli* colicin-producing strain EMO3-C [30] (just called C in the main text) is a derivative of E2^C^-BZB1011 (C_Original_) [1] and carries the pMO3 plasmid encoding the Yellow Fluorescent Protein (YFP) [30]. On this plasmid, the *yfp* gene is under the control of the same promoter as the ColicinE2 operon. Hence, YFP expression correlates directly with ColicinE2 expression and serves as a visual marker for toxin-producing cells. Moreover, in Mader et al. [30], we showed that every cell that produces YFP also lyses. As cell lysis is coupled to toxin expression in this strain, YFP expression can be used as a proxy to assess the number of cells that produce the colicin. The competitor strains S_RFP_, S_NFP_, R_RFP_, and R_NFP_ [3] are derivatives of BZB1011 (S) and E2^R^-BZB1011 (R), respectively [1], and carry plasmids on which the RFP mCherry or NFP is encoded. RFP expression is under the control of the pBAD promoter, which provides for continuous expression in the presence of arabinose. In contrast, YFP in EMO3-C is only expressed when a cell produces colicin. The S_RFP_ and S_NFP_ strains are sensitive to the toxin, due to the lack of the original colicinE2-producing pColE2-P9 plasmid that also encodes an immunity protein protecting the otherwise genetically identical C strain from ColicinE2-induced DNA damage. The strains R_RFP_, and R_NFP_ are resistant towards the toxin due to impaired colicin uptake [41]. Please note that the expression of the RFP mCherry impairs an additional metabolic cost and hence reduces growth rates relative to the NFP strains [3] (S7A Fig). In contrast, the expression of YFP (in EMO3-C) does not considerably affect the growth rate of this strain (growth rate in liquid media [see below for calculation] 0.735 ± 0.47 1/h for E2^C^-BZB1011 (C_Original_) and 0.728 ± 0.026 1/h for EMO3-C). The respective fluorescence proteins expressed in the S strain (S_RFP_) and the C strain (YFP) were chosen to ensure a similar growth rate of these strains in the absence of an external stressor (Fig 3B).

Strain S_YFP_ [30] is a derivative of BZB1011 (S) [1]. It lacks the pColE2-P9 plasmid but carries the plasmid pMO2 [30]. Similar to pMO3, this plasmid encodes YFP, with the *yfp* gene being under the control of the same promoter as the ColicinE2 operon. Hence, YFP expression in S_YFP_ correlates directly with ColicinE2 expression, although this strain is unable to produce the toxin due to a lack of original pColE2-P9 plasmid. In contrast to pMO3, pMO2 additionally encodes the gene required for toxin release through cell lysis that is again under the control of the ColicinE2 operon promoter [30]. Hence, this strain is able to lyse upon induction of the ColicinE2 operon promoter, ensuring similar lysis gene expression dynamics as present in EMO3-C. As this strain is not expressing the colicin and has therefore a reduced metabolic cost, it grew slightly faster than EMO3-C, with a growth rate in liquid media of 0.972 ± 0.029 1/h.

Experiments were performed on solid M63 growth medium [42] (1.5% agar) containing 100 μg/ml ampicillin and 0.2% arabinose. Furthermore, the medium was supplemented with the SOS agent MitC at different concentrations (0.0, 0.005, 0.01, 0.1 μg/ml). The growth medium was prepared in rectangular 127.8 x 85.5 mm Greiner OneWell plates (Catalogue Number 670190).

Prior to experiments, bacterial cultures were grown separately in liquid M63 medium, with 100 μg/ml ampicillin and 0.2% arabinose shaken at 300 rpm at 37°C. Overnight cultures were diluted to OD_600_ = 0.1 and grown to OD_600_ = 0.2. The EMO3-C culture was then centrifuged through a 100-kD filter to remove colicin molecules (62 kD) already present in the starter culture. Prior to transfer onto agar, starter cultures were diluted to OD_600_ = 0.1. For competition experiments, EMO3-C cells were mixed with a competitor strain X (S_RFP_, S_NFP_, R_RFP_, or R_NFP_) at a C:X ratio of 1:100 shortly before transfer. This C:X ratio was chosen on the basis of earlier results demonstrating that strain coexistence was only possible at reduced C strain ratios [3]. For control experiments, monoclonal cultures were transferred without mixing.

For experiments with varying initial density, starter cultures were concentrated via centrifugation, diluted to chosen OD_600_ (0.2, 0.4, 0.8) and processed further as described above.

### Phenotypic heterogeneity experiments

To verify known heterogeneities with regard to toxin production by the C strain in liquid environments [30] for the conditions used in our range-expansion experiments, we determined the YFP fluorescence intensity of the C strain on agar plates using a high-resolution setup consisting of a Nikon Eclipse 90i with a Nikon Intensilight light source and a 50× objective operated by Nikon NIS Elements Advanced Research software (version 3.22.01). The system was maintained at 37°C by a custom-built heat box. Starter cultures of the C strain were grown from OD_600_ = 0.05 to 0.2, diluted to OD_600_ = 0.01, and 1-μl droplets were pipetted onto agar plates prepared as described above. The plates were then incubated at 37°C, and images were recorded every 60 min for 5 h in bright-field and fluorescence channels. Applying a fluorescence threshold of 200 fluorescence units, cells were classified into ON and OFF phenotypes (S6 Fig and Fig 3D). Note that these experiments tend to underestimate the producer fraction because already lysed cells were difficult to distinguish from living cells in the OFF state. A minimum of 169 cells (for each data point of Fig 3D) for each inducer concentration was investigated.

### Experiments in liquid culture

Bacterial population growth, the influence of colicinE2 on growth of S, as well as the C–S interaction under well-mixed conditions were analyzed with a plate reader (POLARstar OPTIMA, BMG Labtech). Overnight cultures were generated as described above, diluted to OD_600_ = 0.1 and grown to OD_600_ = 0.2, and for measurements, the cultures were diluted again to OD_600_ = 0.1. Bacterial population growth and interaction was followed for 18 h, while the cultures were maintained at constant shaking at 37°C. Optical density (600 nm) and respective fluorescence channels (YFP and RFP) were measured every 15 min.

After blank correction, the population growth rate (GR) was obtained as follows: Population growth curves as represented by OD_600_ were fitted by using the linear fit function (*f*_*L*_ = *a* +*b* * *x*, with *a* the *y*-intercept and *b* the slope of the function) of the IGOR PRO 6.36 software to fit the natural logarithm of the population growth curves in the exponential growth phase. The population growth rate (GR) was then calculated as: $GR=\frac{b}{ln\left(2\right)}$ for uninduced and 0.005 μg/ml MitC induced samples and averaged.

To test the influence of colicinE2 on the growth of S, the colicin was extracted from the supernatant of a C strain culture induced with 0.7 μg/ml MitC, using 10 K centrifugal filter units (Amicon). The obtained total protein concentration was 1.14 mg/ml. This protein solution was then added to S strain cultures at the concentrations/dilutions indicated in S3D Fig

For C–S interaction experiments under well-mixed conditions, the C and S strain were applied at the 1:100 ratio in accordance to C–S interactions on the solid agar surface.

### Range expansion experiments

Competition experiments were performed over a period of 48 h using the following multiscale set-up, which allows us to monitor up to 77 competition experiments in parallel. A Nikon SMZ 25 stereoscopic microscope with a custom-built mount was assembled on a Newport Isostation table. Image acquisition was performed by a Nikon Qi1 CCD camera controlled by a Nikon DS-U3 camera control unit. A Märzhäuser SCAN 130 x 85 scanning stage controlled via a Märzhäuser TANGO 2 facilitated parallel investigation of multiple communities on the experimental plate. Bright-field illumination and fluorescence excitation were provided by a Lumencor Sola SE II LED lamp. Nikon P2-EFL GFP-B and P2-EFL RFP-L filter blocks were used for fluorescence excitation and emission filtering, and a customized OG-570 long pass filter reduced phototoxicity from bright-field illumination. Microscope, camera, scanning stage, and LED lamp were operated via Nikon NIS-Elements AR 4.30.01 64-bit software with the requisite plug-ins. A gas incubation and heating system for multi-well plates (Ibidi) ensured constant environmental conditions (37°C and 80% humidity).

Aliquots of the inoculum culture were deposited on the experimental plate by a Labcyte Echo 550 Liquid Handler using acoustic droplet ejection [43]. Minimal transferrable volumes of 2.5 nl result in initial bacterial communities of approximately 150 single bacterial cells that are distributed within circular areas of approximately 450 μm in diameter (S1 Fig). ColicinE2 can easily diffuse through the agar plate and an effective toxin range of 100–400 μm [27,44] was described. To prevent touching of neighboring S–C colonies during competition (maximum competition colony size is 4.9 mm [diameter]) and to reduce colicin interactions in between spots to a minimum, the distance between the center of two spots was chosen to be 9 mm. Hence, a minimum of 4 mm unoccupied territory is present at the end of a competition experiment in between two neighboring colonies.

Experiments have been repeated 2–3 times. Only communities containing C cells in the initial colonies were analyzed, resulting in a total of 75, 87, 85, and 87 (0.0, 0.005, 0.01, 0.1 μg/ml MitC) competitions for S–C (Fig 1), 87, 108, 108, 128 competitions for S–X (Fig 5), and 106, 83, 107, 108 competitions for S–S_YFP_ (S9 Fig). See S1 Data, sheet “Number of Observations” for details.

The total time course of each experiment (48 h) was divided into four distinct zoom levels to accommodate bacterial population growth. Settings for lamp intensity, detection gain, or exposure time must be adjusted for each zoom level and are listed in S3 Table. Before the start of the actual experiment, images were taken at every zoom level for background correction purposes at every spot. Acquired images had a resolution of 1280 x 1024 pixels and 12-bit depth.

Please note that the fluorescence data obtained correspond to cells present in the top layer of the colony with contributions of the bacterial layers (up to 200) beneath the top layer. Hence, the fluorescence signal represents an average value over the entire colony depth and is only used to monitor the dominant strain in a particular colony section.

### Image and data processing

We developed our image processing routines with Mathworks MATLAB software (version 2013b). Image processing included image segmentation to isolate the bacterial population from the background and classification to distinguish between different strains based on labelling via fluorescent protein expression (S1C Fig). The use of four different zoom levels made it necessary to use four distinct parameter sets for image segmentation, in order to account for varying object dimensions and image characteristics. Additional complexity arose from the different fluorescence intensities of the fluorescent proteins expressed by the strains used. Consequently, the requirements on the fluorescence analysis varied depending on the strains present. Therefore, parametric fine-tuning was performed to match the outcome of the experiment. Details of the image processing routine can be found in the S1 Text and in S3 Table.

Recorded data curves resulting from image analysis were screened for obvious mis-segmentations and mis-classifications. Identified points were replaced by NaN values to correct the curves. Examples for replaced data points are extinction scenarios, in which the image recognition algorithm could not handle empty images. Finalized data curves are exported and further processed with the statistical programming language R (Version 3.2.3), different R packages, and Matlab (Version 2013b) software. Growth rates of the entire colonies (single strain and mixed S–C colonies) were obtained in the linear area growth regime by linear fitting (S9B Fig).

### Detailed analysis of Phase 1

All competition experiments were screened for presence of C cells in the initial colony. Colonies without C cells were not considered for further analysis. Classification of C cells happened via the presence of a YFP signal, absence of a RFP signal in a cell, and/or were deduced from the effect of secreted toxin at later time points. Then, the positions of each C cell and the colony center were determined. To quantify the initial spatial distribution of C cells, three variables were used. The number of initial C cells *N*_*C*,0_ represents the sum of classified C cells at the beginning of the experiment. From the position of initial C cells ${\vec{x}}_{C,0,\;i}$ with respect to the colony center, the average radial distance of these cells from the center was calculated by the center of mass formula $R_{C,0}=\left|{\vec{x}}_{C,0}\right|=\left|\frac{1}{N_{C,0}}\sum_{i=1}^{N_{C,0}}{\vec{x}}_{C,0,\;i}\right|$. The average distance of C cells from their center of mass was calculated by $D_{C,0}=\left|\frac{1}{N_{C,0}}\sum_{i=1}^{N_{C,0}}\left({\vec{x}}_{C,0,\;i}-{\vec{x}}_{C,0}\right)\right|$ as a measure for the spread of C cells over the colony. In a second step, it was checked whether initial C cells grow into viable clusters that reach the colony edge during the first phase of competition or die early due to toxin production and concomitant lysis. The number of these viable C cell clusters at the colony edge after 12 h was aggregated in the variable *N*_*C*,*Edge*_.

Similarly, simulations with 16 different fixed initial conditions *IC* were repeated 30 times each, for the whole range of *s*_*C*_ values (*s*_*C*_ = 0.02 example in S8C Fig). Again, the number of viable C clusters at the colony boundary at the end of the initial phase *N*_*C*,*Edge*_ was determined.

### Statistical analysis

For the experimental data, the influence of *N*_*C*,0_, *R*_*C*,0_, *D*_*C*,0_, and *MitC* on *N*_*C*,*Edge*_ was modelled after standardization using the following linear model.

$$N_{C,Edge}=\beta_{0}+\beta_{1}N_{C,0}+\beta_{2}R_{C,0}+\beta_{3}D_{C,0}+\beta_{4}MitC$$

For both experiment and simulation, the influence of the different factors *MitC*, *N*_*C*,*Edge*_, *N*_*C*,0_, *D*_*C*,0_, and *R*_*C*,0_ on final C fraction *F*_*C*_ was analyzed using a linear model that also incorporated interactions between these factors. For the experimental analysis, *MitC* was treated as a categorical variable to account for the qualitative difference between different induction regimes. The other variables were treated as continuous variables. The model had the explicit form:

$$\begin{array}{l} F_{C}=\beta_{0}+MitC\left(\beta_{1}+\beta_{6}N_{C,Edge}+\beta_{7}N_{C,0}+\beta_{8}D_{C,0}+\beta_{9}R_{C,0}\right)\\ \;+N_{C,Edge}\left(\beta_{2}+\beta_{10}N_{C,0}+\beta_{11}D_{C,0}+\beta_{12}R_{C,0}\right)\\ \;+N_{C,0}\left(\beta_{3}+\beta_{13}D_{C,0}+\beta_{14}R_{C,0}\right)+D_{C,0}\left(\beta_{4}+\beta_{15}R_{C,0}\right)+\beta_{5}R_{C,0} \end{array}$$

Analysis of the simulation was performed analogously using the three variables *s*_*C*_, *N*_*C*,*Edge*_, and *IC*. Here all three variables were treated as categorical variables. The linear model had the explicit form:

$$F_{C}=\beta_{0}+s_{C}\left(\beta_{1}+\beta_{4}N_{C,Edge}+\beta_{5}IC\right)+N_{C,Edge}\left(\beta_{2}+\beta_{6}IC\right)+\beta_{3}IC$$

Significance and effect sizes of the factors were analyzed using the Anova and EtaSq functions of the statistical programming language R (Version 3.2.3) that rely on the type I (sequential) sum of squares, where the order of terms in the model matters. Terms in the model are ordered according to the experimental design: *MitC*/*s*_*C*_ is externally tuned, *N*_*C*,*Edge*_ is found to be very important, exact spatial details/*IC* are of minor importance.

### Numerical modelling

A stochastic lattice-based computational model was used to simulate competition between the C strain and a competitor strain X (X being S_RFP_, S_NFP_, R_RFP_, or R_NFP_). The model incorporated spatial coarse-graining steps during the simulation in analogy to the zooming used in the experimental procedure. Once the simulated expanding colony reached the boundaries of the 250 x 250 pixel lattice, the current colony was coarse-grained by a factor of five, resulting in a coarser 50 x 50 lattice that was positioned in the middle of a new 250 x 250 lattice. Reaction rates were adjusted accordingly. Zooming did not only accommodate the microscopic details of the initial conditions but also permitted simulations to be completed in feasible computational times. Initial communities used for simulations were created in accordance with experimental conditions, having random spatial distributions of C and X cells in an approximate 1:100 (C:X) ratio within a circular field approximately 450 μm in diameter, with at least one initial C cell. Initial colony density was chosen in accordance with experimental conditions (S4 Table).

Five different species of agents were used in this model: viable C and X cells, colicin-producing C_on_ cells, growth-inhibited X_stop_ cells, and unoccupied agar sites A. Reactions were modelled using a Moore neighborhood (eight nearest neighbors), in which the rates for diagonal growth were scaled by a factor of $1/\sqrt{2}$. Possible reactions comprised reproduction of viable C and X cells, C cells switching to a producing C_on_ state, subsequent lysis of the C_on_ cell with concomitant colicin release, and transition to a growth-inhibited X_stop_ state by S cells in response to the action of colicin (Fig 4B). As soon as colicin was released by a lysing C_on_ cell, an exponential colicin profile was assumed to originate from this position, as performed in previous studies [3]. Rates for these reactions are given in S2 Table.

Growth rates, r, were obtained by fitting simulated single-strain curves to experimental control data for the S strain without induction by MitC and assuming a simple linear relationship between area growth and microscopic growth rates [45] (S2 Table, S9B Fig). The lysis rate of C_on_ cells adopted here was suggested by earlier experimental findings [30]. The C strain’s switching rate to the producing phenotype *s*_*C*_ was chosen such that it covered a broad range of producer fraction values (S4 Table). Assuming a steady state for the toxin producer dynamics $\partial_{t}C_{on}=s_{C}\cdot C-d_{C_{on}}\cdot C_{on}$, the relationship between switching rate *s*_*C*_, the lysis rate of toxin producers **$d_{C_{on}}$**, and the number of reproducing C and toxin-producing C_on_ cells can be approximated by $\frac{C_{on}}{C}=\frac{s_{C}}{d_{C_{on}}}$. The remaining free parameter, toxin effectivity *s*_*X*_ = *σ*_*x*_ ⋅ *n*_*tox*_, is composed of two terms, toxin sensitivity of the competitor strain *σ*_*x*_ and toxin amount factor *n*_*tox*_ representing the amount of toxin produced by *C*_*on*_. Because the toxin effectivity parameter was hard to determine experimentally, the system was simulated for a range of values, testing for robustness and dependence on this parameter (Fig 4C, S8A Fig, S4 Table). A toxin sensitivity of *σ*_*x*_ = 1,500 and toxin amount factor *n*_*tox*_ = 1.0 were chosen as “standard conditions.”

For simulations of the impact of growth rate variation, a fixed switching rate (*s*_*C*_ = 0.015) and fixed C strain growth rate were used in combination with a range of toxin sensitivities and relative competitor growth rates and a fixed toxin amount factor *n*_*tox*_ = 1.0 (S4 Table). For every *s*_*X*_, *s*_*C*_ or *r*_*X*_, *s*_*C*_ pair simulations were repeated 48 times.

Besides the purely stochastic simulations, we performed simulations in which synchronous toxin production and release was explicitly implemented. In these simulations, the C strain grew without switching to the producing state for 50 min and did not replicate for a further 50 min, at which point all cells produced and released their toxin simultaneously (synchronicity scenario I, S8B Fig). Additionally, a second synchronicity scenario was implemented where C cells reproduced for 100 min without switching to the producing state, prior to producing and releasing the toxin collectively (synchronicity scenario II, S8B Fig).

## Supporting information

S1 Fig Experimental approach and analysis. (A) Bacteria are transferred to solid growth medium by acoustic droplet ejection. (B) Bacterial communities are observed in multiple parallel experiments. An incubation chamber ensures constant environmental conditions. (C) Demonstration of segmentation into background and bacterial areas and classification of segmented area according to bacterial strains. (D&E) Comparison of the same bacterial cells imaged with two different set-ups shows the ability of the multi-scale stereo microscope (D) to detect single cells. However, it is clear that cells that are in close proximity to each other cannot be resolved as well as with a high-resolution upright microscope (E).
(TIF)

S2 Fig Temporal evolution of the interaction between the S strain (magenta) and the C strain (green) resulting in different outcomes. Rows A, D, E, and F depict experiments performed with 0.01 μg/ml MitC, row B and C with 0.0 μg/ml MitC. Green circles highlight the positions of the initial toxin producers. (A) All three C cells release their toxin and die. The toxin kills sensitive cells in the vicinity but cannot prevent the S strain from prevailing in the long run. (B) Of two initial C cells one switches into the producing state early, killing neighboring S cells. The second C cell does not switch to the producing state, replicates, and forms a viable population that coexists with the S strain. (C) Coexistence between C and S with three C strain clusters. (D) A single C cell is barely identifiable during the first hours of the experiment, but is able to develop into a viable population that produces enough toxin to halt sensitive strain’s growth and subsequently dominates the population. (E) Two of the three initial C cells respond quickly to the external stress, produce the toxin, and kill nearly all sensitive cells. That enables the remaining C to take over the population. (F) Two initial C cells produce toxin immediately and kill all sensitive cells quickly. That leads to extinction of the whole population.
(TIF)

S3 Fig Effect of ColicinE2 release on growth of S under well-mixed conditions. (A) Growth of the C population in liquid C-S competition experiments over time as given by YFP expression (Methods) for four different MitC concentrations. (B) Growth of the S population in liquid C-S competition experiments over time as given by RFP (mcherry) expression (Methods) for four different MitC concentrations. S growth is arrested (saturation in RFP value) as soon as a considerable amount of C cells (increase in C strain population) is present releasing the toxin (see A). (C) Final FI values of C and S strain populations in liquid C-S competition experiments at four different MitC concentrations. While the S strain population (magenta) decreases with MitC concentration due to increased toxin release by C, the C strain population reaches maximum values at intermediate MitC concentrations (green). (D) Growth of S in liquid medium, in the presence of different sub-lethal concentrations of extracted ColicinE2 supernatant (Methods). Low MitC concentrations (0–0.0001 dilutions) do only slightly decrease growth of the S strain population. At an intermediate concentration of 0.001 dilution MitC growth is initially strongly reduced, but increases at later time-points leading to higher maximum growth values as obtained for the S strain population grown in the absence of MitC. At high inducer concentration (0.01 dilution) growth of the S strain population is completely inhibited. Data represent average values over at least 5 experiments.
(TIF)

S4 Fig Influence of initial C strain distribution and number on competition outcome. (A,B) Experimental observations: Neither initial position of the C strain (A) nor variations in the initial C strain numbers (B) have an impact on competition outcome. Colors of the single dots indicate the outcome of an experiment (e.g. coexistence in dark grey). The color code is given on the right. (A) Initial spatial distribution of individual C cells (single dots) in each competition experiment is shown for different inducer concentrations. Single dots represent all initial C cells in all analyzed competition experiments. (B) The final fraction of the C strain after 48 h of interaction is plotted against initial C cell number in each competition experiment, for different MitC concentrations. (C) Theoretical analysis of 16 initial conditions *IC* (initial C strain distribution, x-axis) repeated 30 times each is shown for one exemplary switching rate (s_C_ = 0.02). The data show varying competition outcomes (F_C_, fraction of C strain after 48hrs, y-axis) for the very same initial condition.
(TIF)

S5 Fig Initial density variation for S-C competition in experiment and simulation. Top row: Initial colony examples for different initial densities. Middle row: Experimental outcome distribution of competitions with 0.005μg/ml MitC. Bottom row: Simulated competition outcome distributions (s_C_ = 0.031853929) with initial densities corresponding to experimental conditions.
(TIF)

S6 Fig The fraction of C cells producing and releasing the toxin Colicin E2 increases with inducer concentration. High-resolution snapshots of C populations after 100 min of growth reveal that the fraction of colicin-producing (YFP) C cells rises with increasing mitomycin C concentrations.
(TIF)

S7 Fig Importance of division of labor for C strain success. All-ON or all-OFF scenarios do not allow for C strain dominance. (A) control interaction experiments with a toxin deficient but otherwise identical strain S_YFP_ with the S_RFP_ strain, show that without toxin release the S_YFP_ strain cannot outcompete the S_RFP_ strain but only suffers from lysis with increased inducer concentration instead. (B) In a scenario where induction was increased to induce all C cells, C success could not be observed anymore, instead we find mostly extinction. (C) Simulated competition outcome distributions. Normal simulation with active lysis, 50% toxin producer fraction within the C strain, and toxin amount *S_S_* of 1500 (left). Simulation where all C cells (toxin producer fraction 100%) do not lyse, but release their toxin upon replication. However replication rate and toxin amount where half as much as in the ordinary simulation (right).
(TIF)

S8 Fig Phase diagrams and extinction outcome obtained by computational modeling. (A) Phase diagrams for toxin effectivity s_S_ and toxin producer fraction show C dominance for intermediate producer fractions at various values of s_S_. (B) Outcome distribution of experiments, minimal model simulation, and two variants of synchronous toxin production and release simulations (Methods) show different probabilities of extinction outcomes.
(TIF)

S9 Fig Determination and influence of growth rate on competition outcomes. (A) Growth rates obtained in control experiments at 0.005 μg/ml MitC for the different competitor strains used. (B) Relationship between average area curves of the S strain at 0.0 μg/ml MitC in control experiments (black x’s), linear fit of linear growth regime (red) and optimized computational trajectory (blue). (C) C success, S success, coexistence and extinction phase diagrams for simulations with different growth rates. Black rectangles indicate the distributions used in Fig 5.
(TIF)

S1 Table Linear regression ANOVA results of initial conditions on final C strain fraction. (TIF)

S2 Table Area growth rates obtained in experiments and model growth rates for simulation. (TIF)

S3 Table Experimental settings and image processing parameters used. (TIF)

S4 Table Parameters used in the theoretical modeling. (TIF)

S1 Text Image analysis details. (DOCX)

S1 Data Supplementary data file. Supplementary Excel data file containing all data used to generate figures and tables and additional statistical information.
(XLSX)

S1 Video Experimental competition: S wins. Time-lapse recording of competition experiment shows success of S strain (magenta) after toxin producing C cells kill S cells in the upper part of the colony. See S2A Fig for details.
(AVI)

S2 Video Experimental competition: Coexistence. Time-lapse recording of competition experiment shows coexistence of C strain (green) with S strain (magenta).
(AVI)

S3 Video Experimental competition: C wins. Time-lapse recording of competition experiment shows success of C strain (green).
(AVI)

S4 Video Experimental competition: Extinction. Time-lapse recording of competition experiment shows extinction both strains.
(AVI)

S5 Video Model competition: S wins. Simulation shows success of S strain. Color code: Viable S cells (bright red), growth inhibited S cells (dark red), reproducing C cells (blue), and toxin producing C cells (green). (Simulation parameters: *s_S_* = 1500, *s_C_* = 0.002581).
(AVI)

S6 Video Model competition: Coexistence. Simulation shows coexistence of both strains. Color code: Viable S cells (bright red), growth inhibited S cells (dark red), reproducing C cells (blue), and toxin producing C cells (green). (Simulation parameters: *s_S_* = 1500, *s_C_* = 0.002581).
(AVI)

S7 Video Model competition: C wins. Simulation shows success of C strain. Color code: Viable S cells (bright red), growth inhibited S cells (dark red), reproducing C cells (blue), and toxin producing C cells (green). (Simulation parameters: *s_S_* = 1500, *s_C_* = 0.02).
(AVI)

S8 Video Model competition: Extinction. Simulation shows extinction of both strains. Color code: Viable S cells (bright red), growth inhibited S cells (dark red), reproducing C cells (blue), and toxin producing C cells (green). (Simulation parameters: *s_S_* = 1500, *s_C_* = 0.155).
(AVI)

S9 Video Model competition: Synchronicity scenario I. Simulation shows extinction of both strains after replication of the C strain for 50 minutes, followed by 50 minutes of pausing before all C cells release the colicin. Color code: Viable S cells (bright red), growth inhibited S cells (dark red), reproducing C cells (blue), and toxin producing C cells (green). (Simulation parameters: *s_S_* = 1500).
(AVI)

S10 Video Model competition: Synchronicity scenario II. Simulation shows extinction of both strains after replication of the C strain for 100 minutes, followed by immediate release of colicin by all C cells. Color code: Viable S cells (bright red), growth inhibited S cells (dark red), reproducing C cells (blue), and toxin producing C cells (green). (Simulation parameters: *s_S_* = 1500).
(AVI)

## Abbreviations

C strain: ColicinE2 producer strain
mitC: mitomycin C
NFP: no fluorescing protein
RFP: red fluorescent protein
S strain: toxin-sensitive strain
YFP: yellow fluorescent protein
