# Supplementary figures and images for: Effects of stochasticity and division of labor in toxin production on two-strain bacterial competition in Escherichia coli
Source: PLoS Biol. 2017 May 1;15(5):e2001457. doi: 10.1371/journal.pbio.2001457 (PMC5411026; doi:10.1371/journal.pbio.2001457)

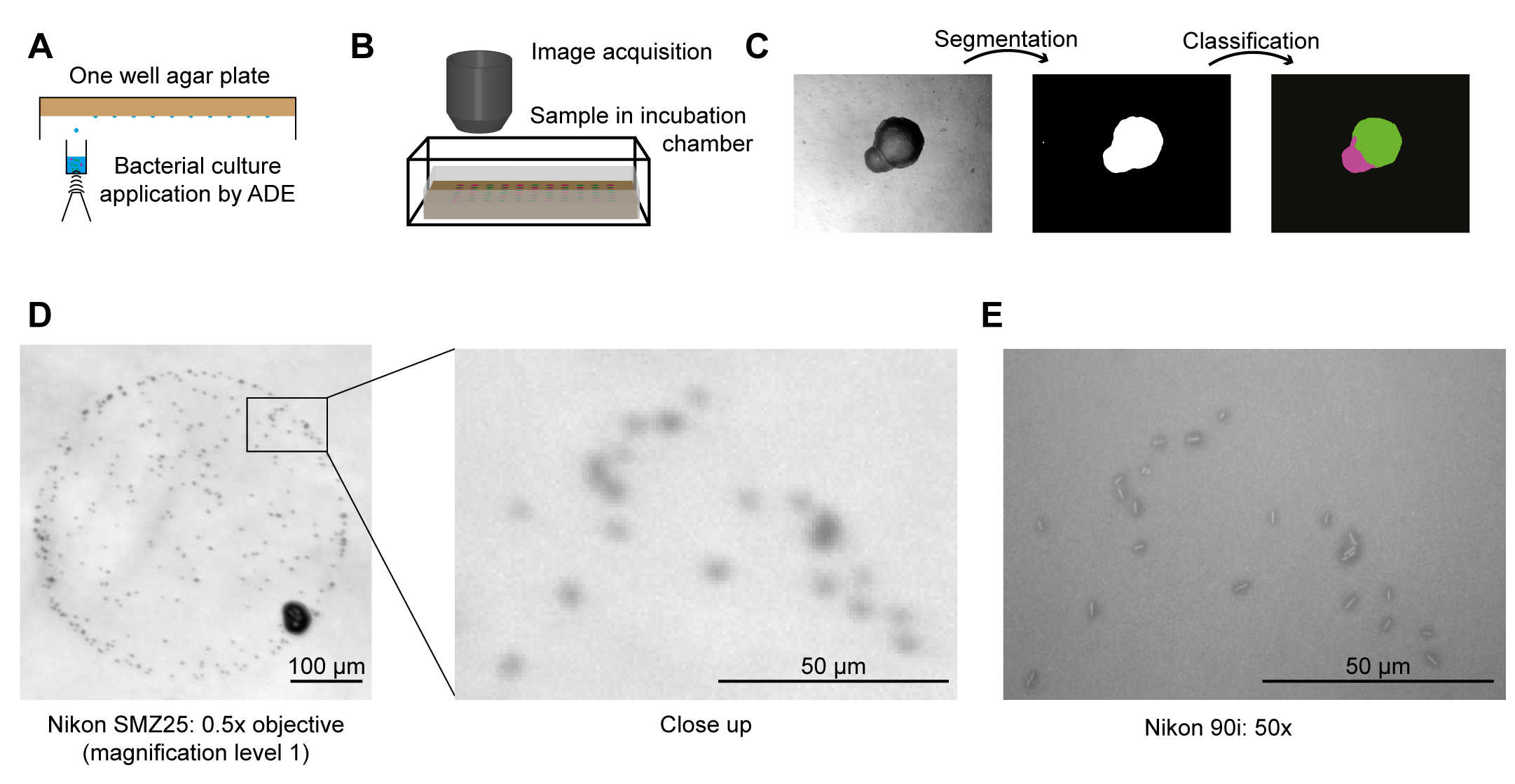

Supplement: S1 Fig — (A) Bacteria are transferred to solid growth medium by acoustic droplet ejection. (B) Bacterial communities are observed in multiple parallel experiments. An incubation chamber ensures constant environmental conditions. (C) Demonstration of segmentation into background and bacterial areas and classification of segmented area according to bacterial strains. (D&E) Comparison of the same bacterial cells imaged with two different set-ups shows the ability of the multi-scale stereo microscope (D) to detect single cells. However, it is clear that cells that are in close proximity to each other cannot be resolved as well as with a high-resolution upright microscope (E). (TIF) [file pbio.2001457.s001.tif]

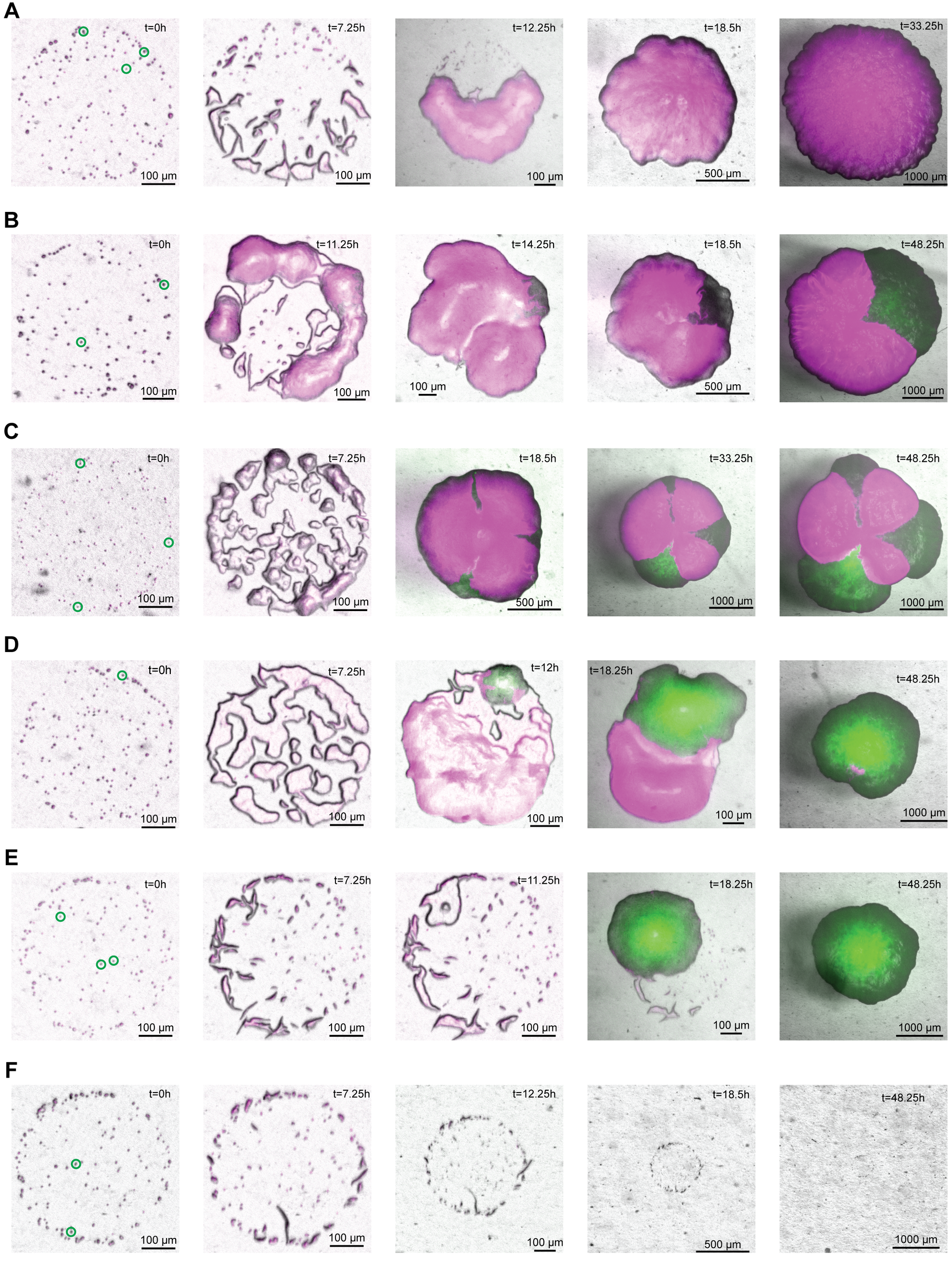

Supplement: S2 Fig — Rows A, D, E, and F depict experiments performed with 0.01 μg/ml MitC, row B and C with 0.0 μg/ml MitC. Green circles highlight the positions of the initial toxin producers. (A) All three C cells release their toxin and die. The toxin kills sensitive cells in the vicinity but cannot prevent the S strain from prevailing in the long run. (B) Of two initial C cells one switches into the producing state early, killing neighboring S cells. The second C cell does not switch to the producing state, replicates, and forms a viable population that coexists with the S strain. (C) Coexistence between C and S with three C strain clusters. (D) A single C cell is barely identifiable during the first hours of the experiment, but is able to develop into a viable population that produces enough toxin to halt sensitive strain’s growth and subsequently dominates the population. (E) Two of the three initial C cells respond quickly to the external stress, produce the toxin, and kill nearly all sensitive cells. That enables the remaining C to take over the population. (F) Two initial C cells produce toxin immediately and kill all sensitive cells quickly. That leads to extinction of the whole population. (TIF) [file pbio.2001457.s002.tif]

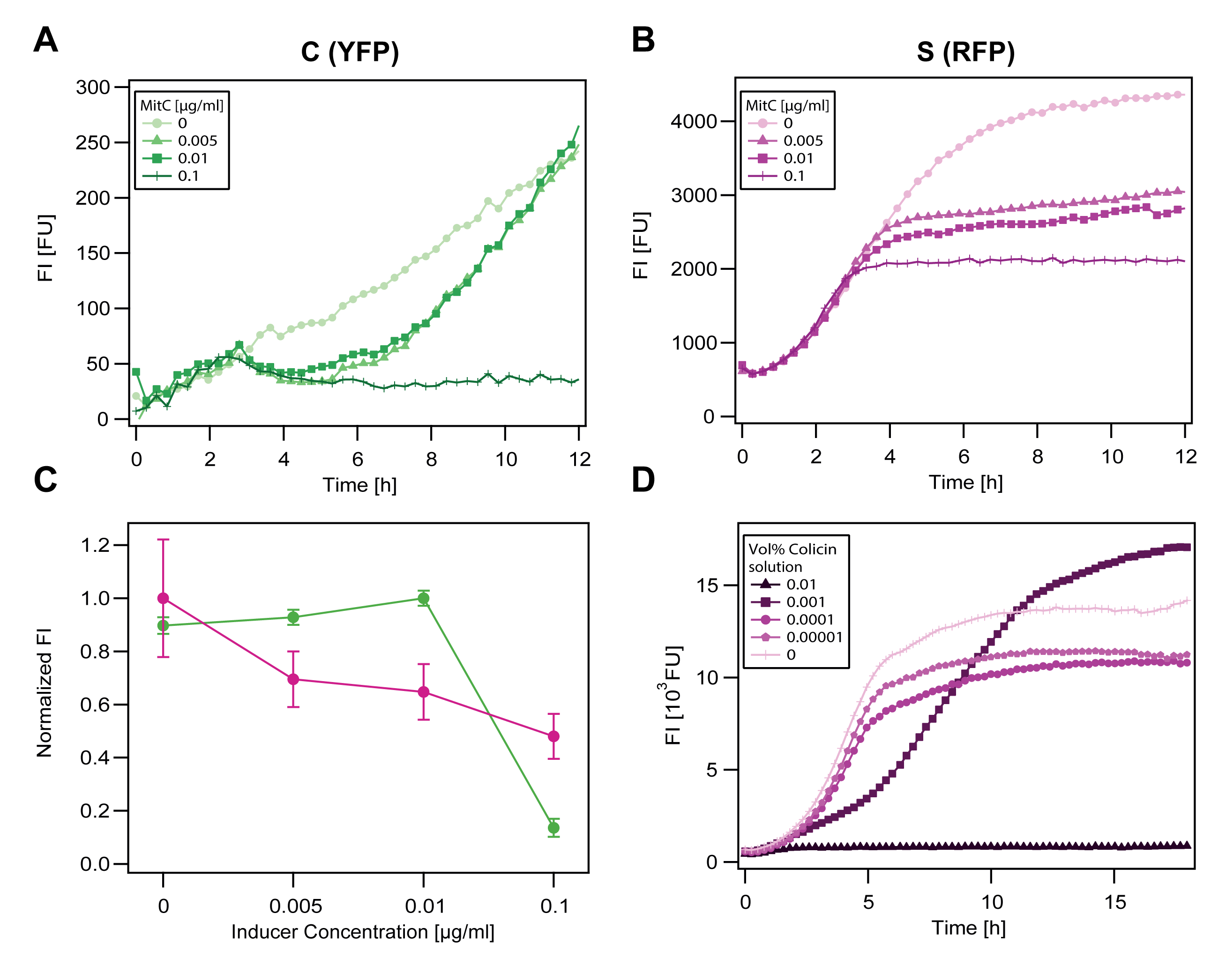

Supplement: S3 Fig — (A) Growth of the C population in liquid C-S competition experiments over time as given by YFP expression (Methods) for four different MitC concentrations. (B) Growth of the S population in liquid C-S competition experiments over time as given by RFP (mcherry) expression (Methods) for four different MitC concentrations. S growth is arrested (saturation in RFP value) as soon as a considerable amount of C cells (increase in C strain population) is present releasing the toxin (see A). (C) Final FI values of C and S strain populations in liquid C-S competition experiments at four different MitC concentrations. While the S strain population (magenta) decreases with MitC concentration due to increased toxin release by C, the C strain population reaches maximum values at intermediate MitC concentrations (green). (D) Growth of S in liquid medium, in the presence of different sub-lethal concentrations of extracted ColicinE2 supernatant (Methods). Low MitC concentrations (0–0.0001 dilutions) do only slightly decrease growth of the S strain population. At an intermediate concentration of 0.001 dilution MitC growth is initially strongly reduced, but increases at later time-points leading to higher maximum growth values as obtained for the S strain population grown in the absence of MitC. At high inducer concentration (0.01 dilution) growth of the S strain population is completely inhibited. Data represent average values over at least 5 experiments. (TIF) [file pbio.2001457.s003.tif]

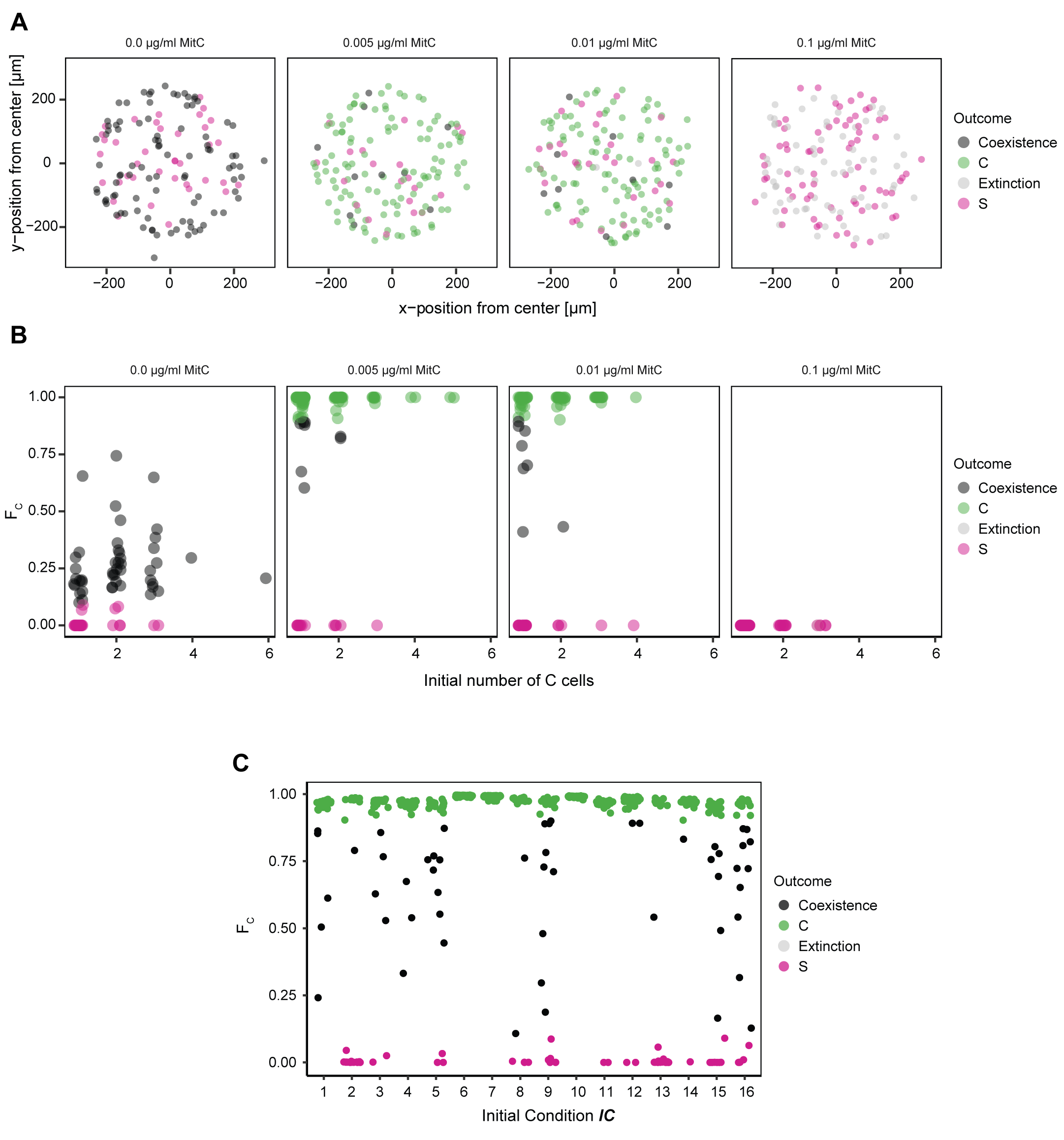

Supplement: S4 Fig — (A,B) Experimental observations: Neither initial position of the C strain (A) nor variations in the initial C strain numbers (B) have an impact on competition outcome. Colors of the single dots indicate the outcome of an experiment (e.g. coexistence in dark grey). The color code is given on the right. (A) Initial spatial distribution of individual C cells (single dots) in each competition experiment is shown for different inducer concentrations. Single dots represent all initial C cells in all analyzed competition experiments. (B) The final fraction of the C strain after 48 h of interaction is plotted against initial C cell number in each competition experiment, for different MitC concentrations. (C) Theoretical analysis of 16 initial conditions IC (initial C strain distribution, x-axis) repeated 30 times each is shown for one exemplary switching rate (sC = 0.02). The data show varying competition outcomes (FC, fraction of C strain after 48hrs, y-axis) for the very same initial condition. (TIF) [file pbio.2001457.s004.tif]

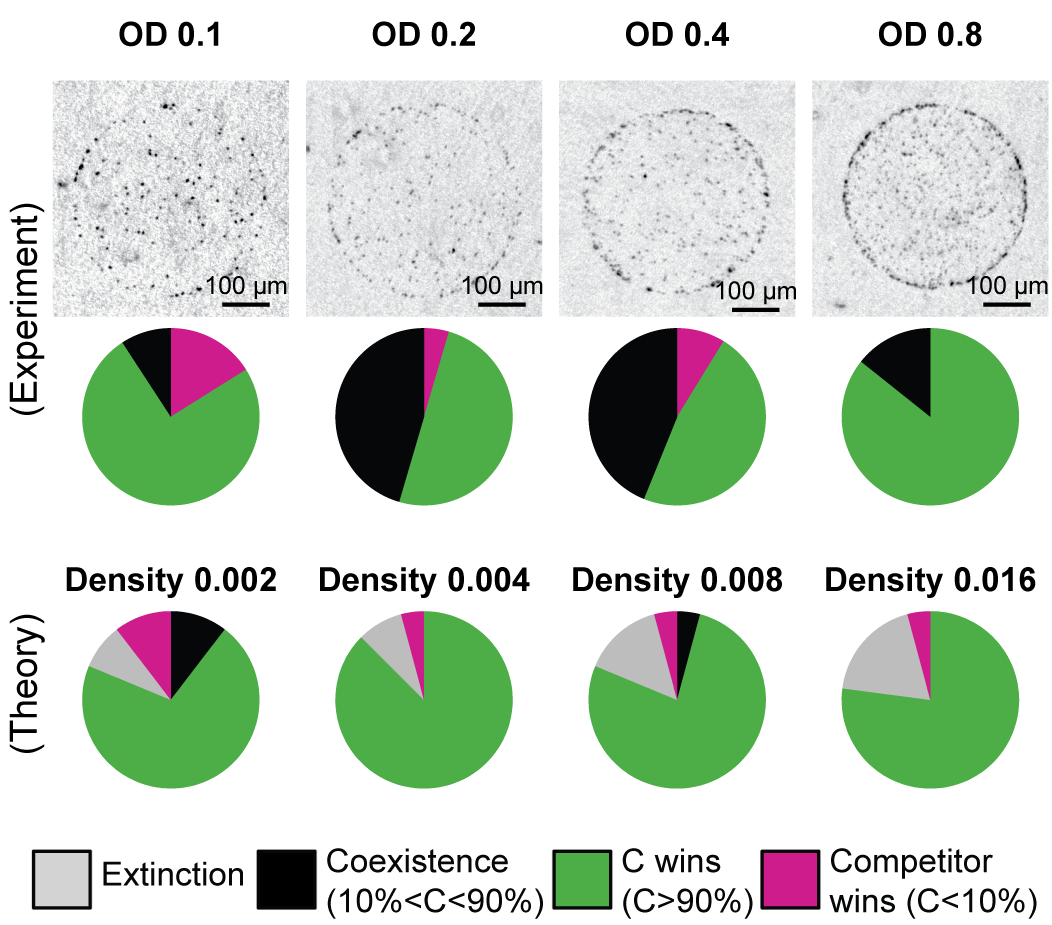

Supplement: S5 Fig — Top row: Initial colony examples for different initial densities. Middle row: Experimental outcome distribution of competitions with 0.005μg/ml MitC. Bottom row: Simulated competition outcome distributions (sC = 0.031853929) with initial densities corresponding to experimental conditions. (TIF) [file pbio.2001457.s005.tif]

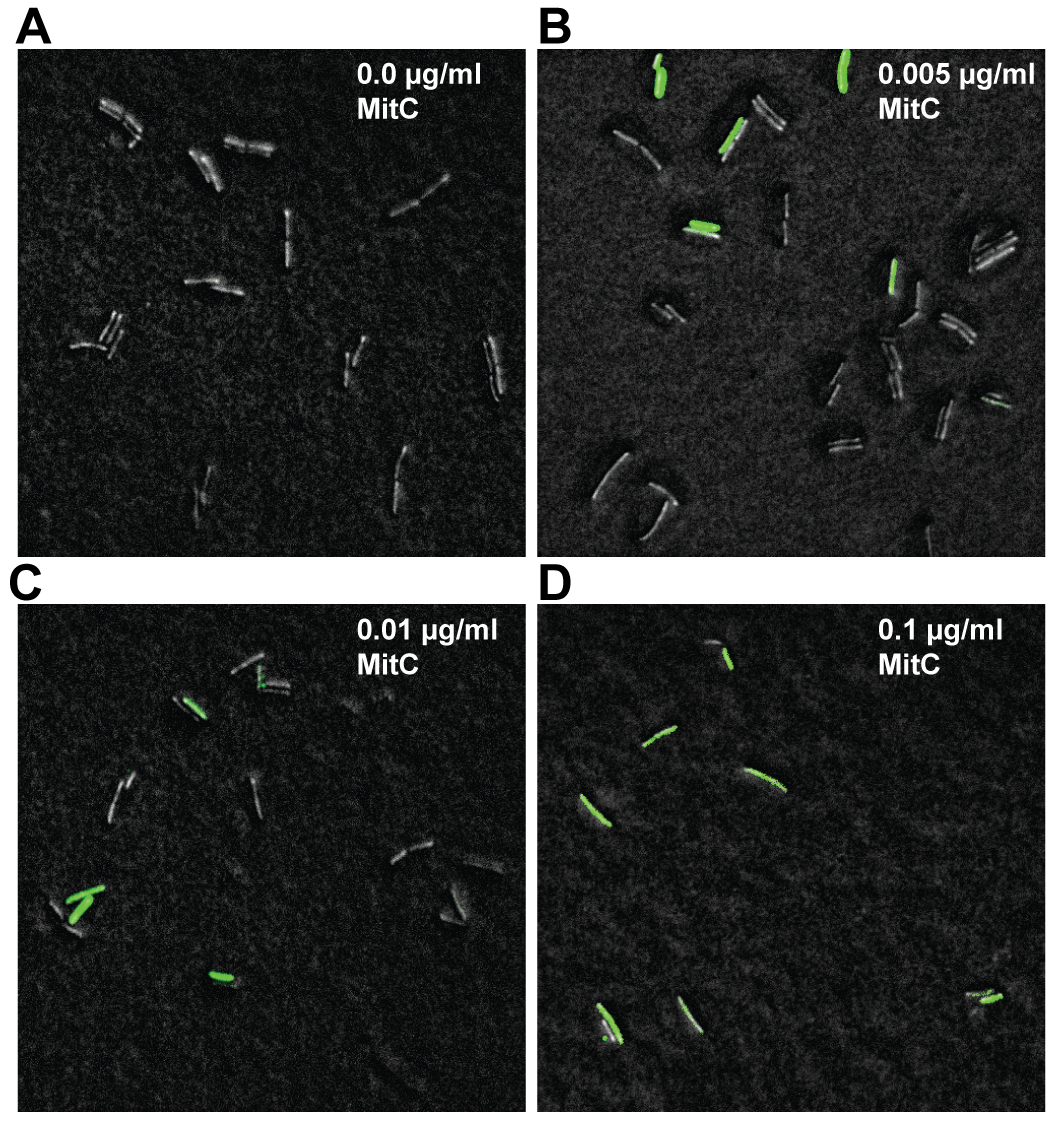

Supplement: S6 Fig — High-resolution snapshots of C populations after 100 min of growth reveal that the fraction of colicin-producing (YFP) C cells rises with increasing mitomycin C concentrations. (TIF) [file pbio.2001457.s006.tif]

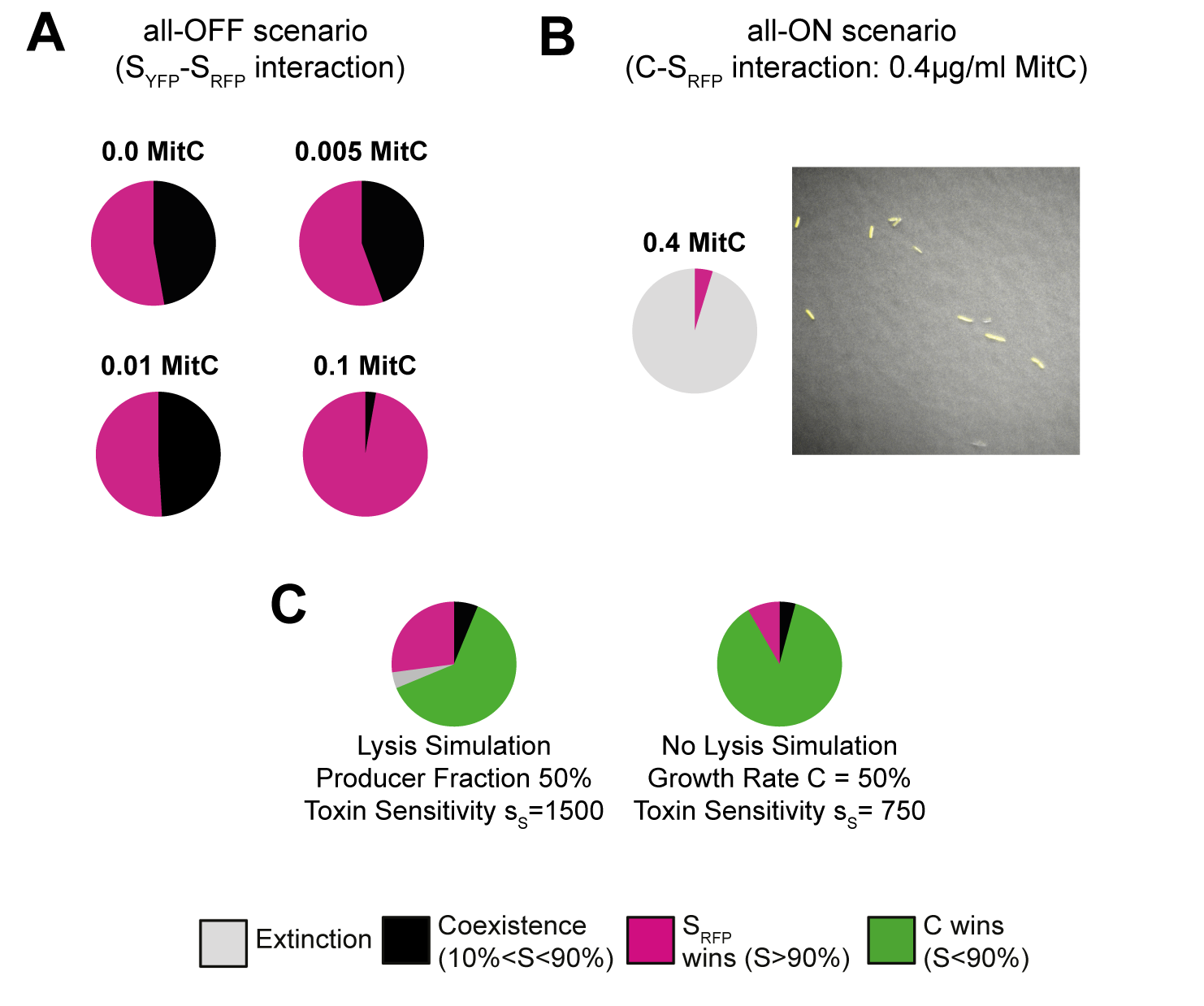

Supplement: S7 Fig — All-ON or all-OFF scenarios do not allow for C strain dominance. (A) control interaction experiments with a toxin deficient but otherwise identical strain SYFP with the SRFP strain, show that without toxin release the SYFP strain cannot outcompete the SRFP strain but only suffers from lysis with increased inducer concentration instead. (B) In a scenario where induction was increased to induce all C cells, C success could not be observed anymore, instead we find mostly extinction. (C) Simulated competition outcome distributions. Normal simulation with active lysis, 50% toxin producer fraction within the C strain, and toxin amount SS of 1500 (left). Simulation where all C cells (toxin producer fraction 100%) do not lyse, but release their toxin upon replication. However replication rate and toxin amount where half as much as in the ordinary simulation (right). (TIF) [file pbio.2001457.s007.tif]

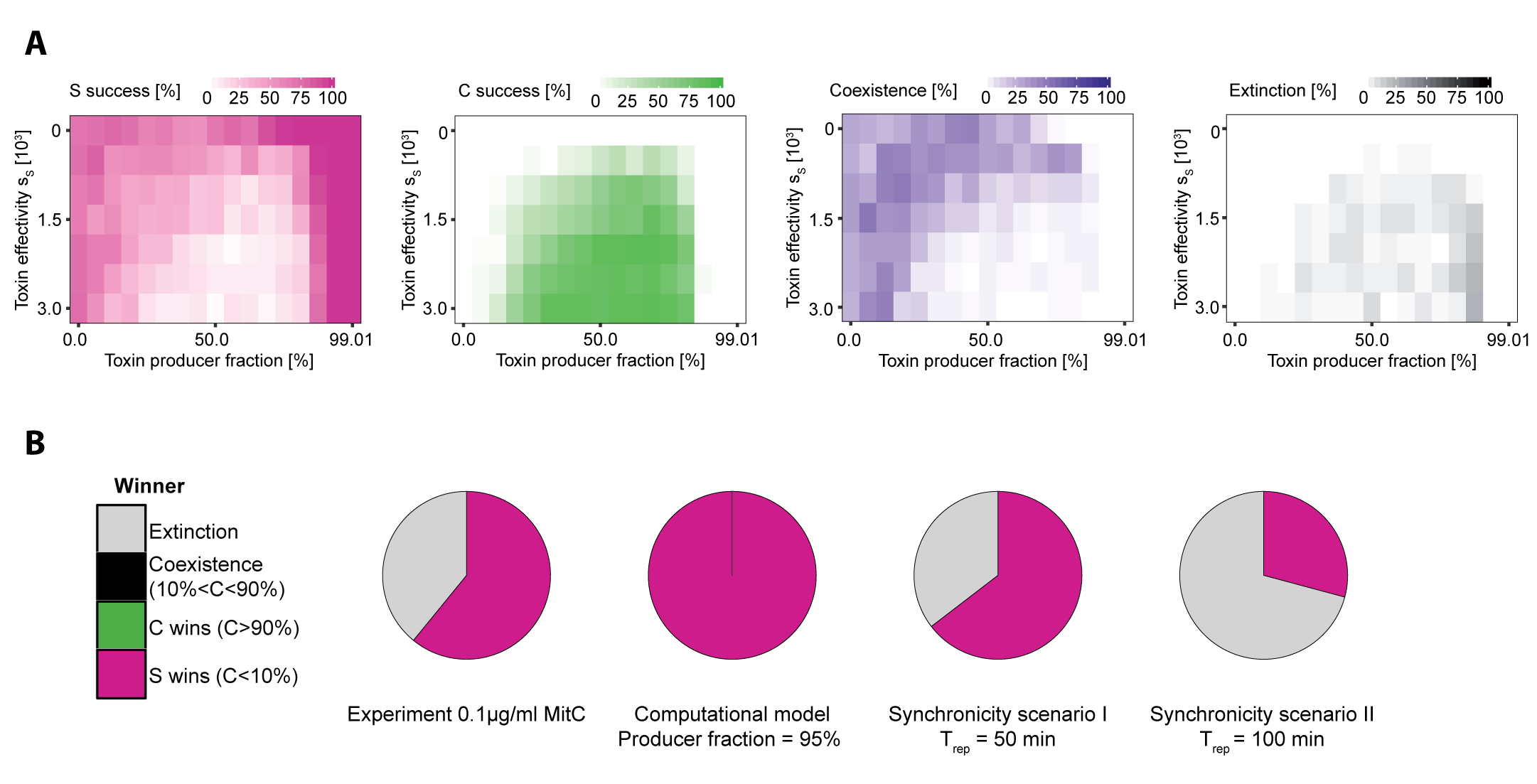

Supplement: S8 Fig — (A) Phase diagrams for toxin effectivity sS and toxin producer fraction show C dominance for intermediate producer fractions at various values of sS. (B) Outcome distribution of experiments, minimal model simulation, and two variants of synchronous toxin production and release simulations (Methods) show different probabilities of extinction outcomes. (TIF) [file pbio.2001457.s008.tif]

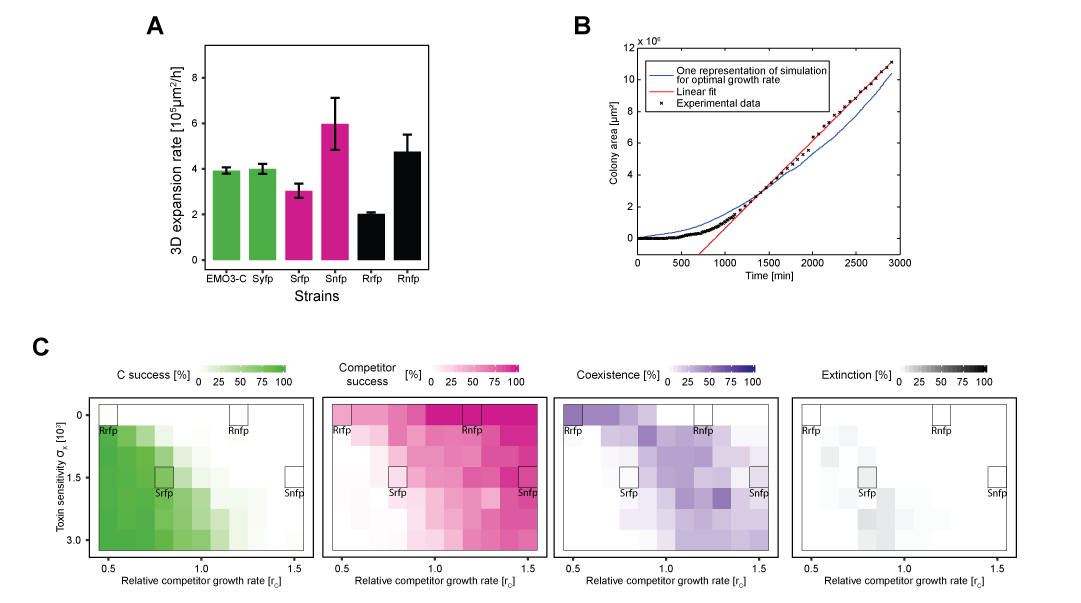

Supplement: S9 Fig — (A) Growth rates obtained in control experiments at 0.005 μg/ml MitC for the different competitor strains used. (B) Relationship between average area curves of the S strain at 0.0 μg/ml MitC in control experiments (black x’s), linear fit of linear growth regime (red) and optimized computational trajectory (blue). (C) C success, S success, coexistence and extinction phase diagrams for simulations with different growth rates. Black rectangles indicate the distributions used in Fig 5. (TIF) [file pbio.2001457.s009.tif]

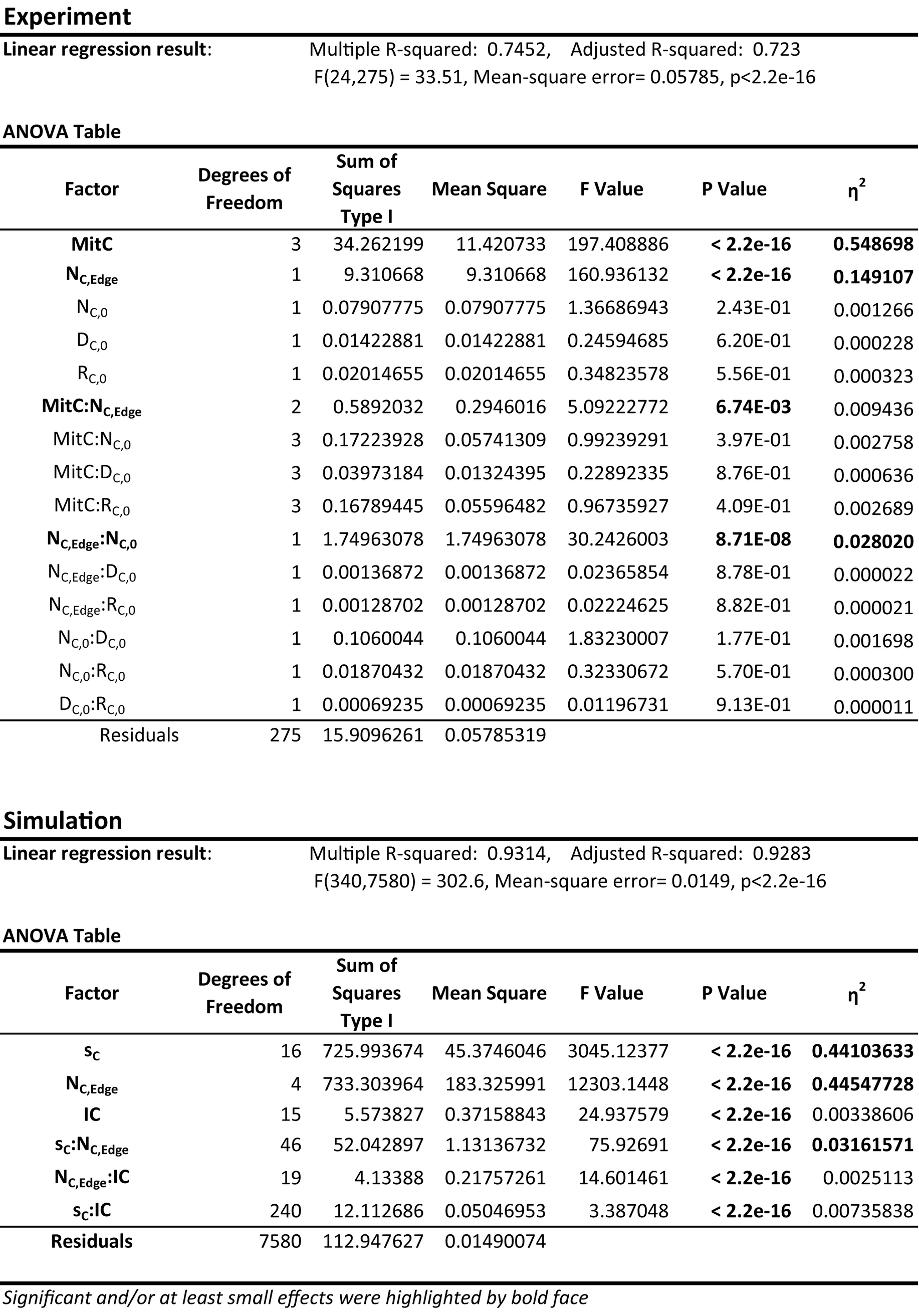

Supplement: S1 Table — (TIF) [file pbio.2001457.s010.tif]

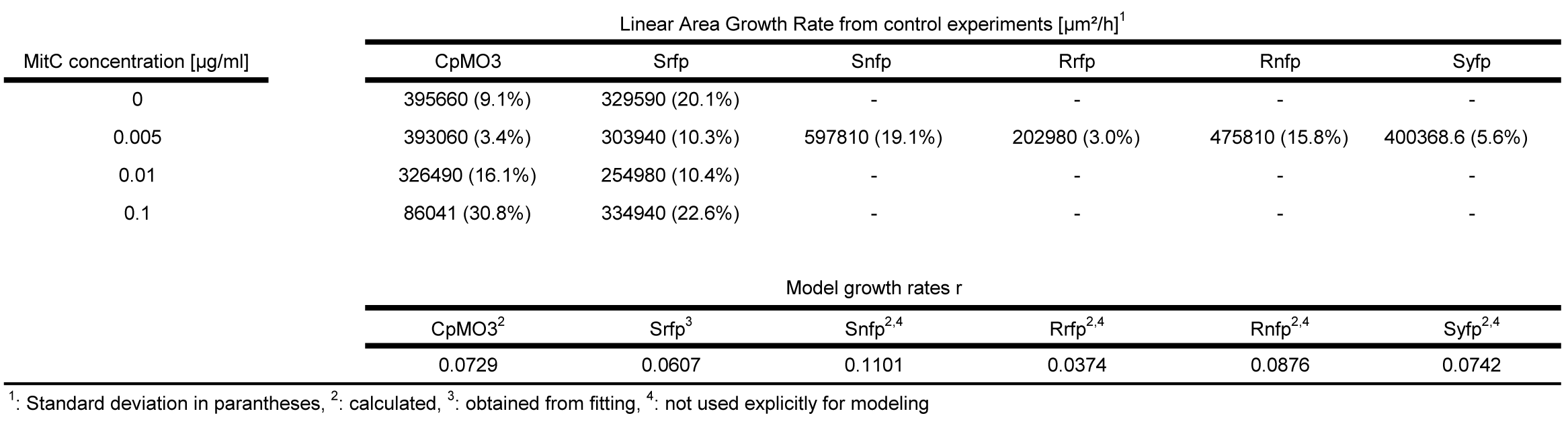

Supplement: S2 Table — (TIF) [file pbio.2001457.s011.tif]

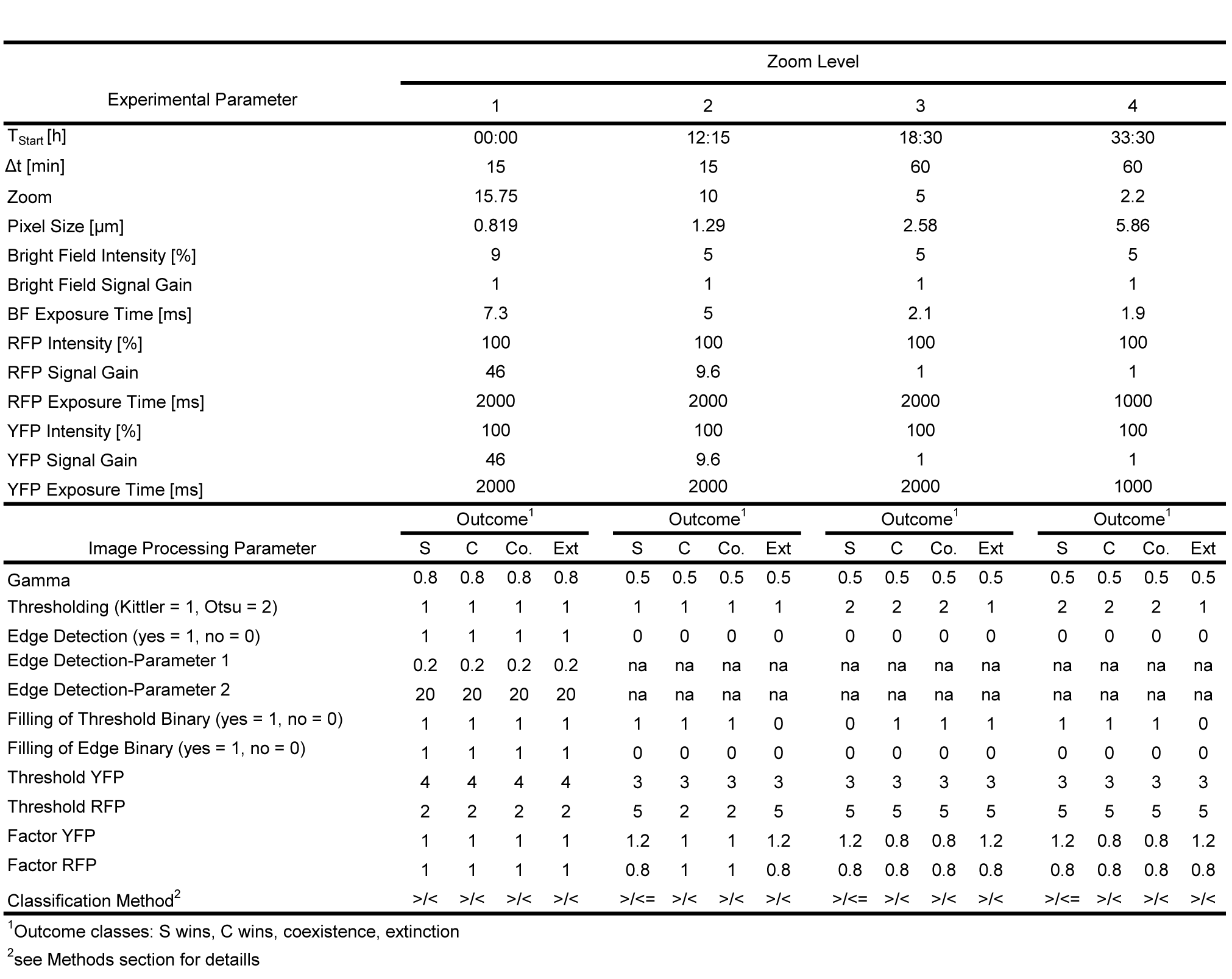

Supplement: S3 Table — (TIF) [file pbio.2001457.s012.tif]

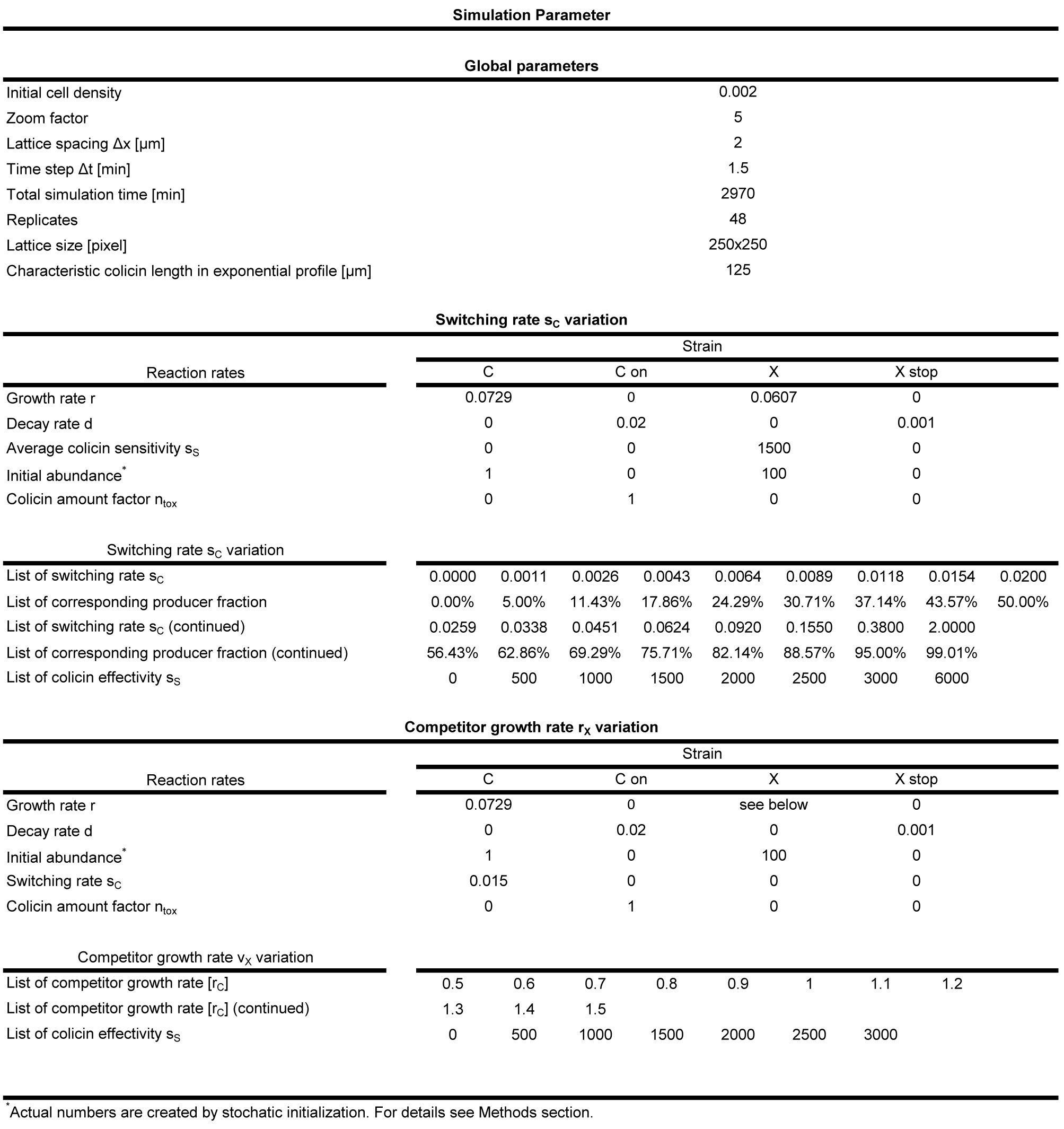

Supplement: S4 Table — (TIF) [file pbio.2001457.s013.tif]
