# Supplementary material for: Effects of stochasticity and division of labor in toxin production on two-strain bacterial competition in Escherichia coli
Source: PLoS Biol. 2017 May 1;15(5):e2001457. doi: 10.1371/journal.pbio.2001457 (PMC5411026; doi:10.1371/journal.pbio.2001457)
Supplement: S1 Text — (DOCX) [file pbio.2001457.s014.docx]

**Supplementary Information Text (S1 Text)**

**Image analysis details**

Preprocessing and image segmentation: All images were converted into 8-bit images. Background images taken on every zoom level before start of each experiment were smoothed with a Gaussian filter (size = 60 pixel, sigma = 60 pixel). Bright-field images were background corrected and inverted by subtracting the original image from the background and taking the absolute value. The intensity values of the resulting images were adjusted (Gamma in S1 Table). Subsequently, noise was reduced by median filtering, and images were corrected for illumination inhomogeneity. Then image segmentation was performed by Kittler-Illingworth thresholding [1] (zoom levels 1 and 2) or by Otsu’s method [2] (zoom levels 3 and 4). In zoom level 1, canny edge detection followed by filling of edges aided in segmentation. The resulting binary images were further processed to fill holes potentially present in connected regions.

Segment classification: The fluorescence image background was determined analogously to the bright field images. Fluorescence image background correction was achieved by subtracting the background from the real image. Then noise was reduced via low-pass filtering, average filtering, and median filtering. The overall median value of the resulting image was calculated, multiplied by a factor (YFP-, RFP-factor in S3 Table) and subtracted from the image. After another noise reduction step by Wiener filtering, the image was adjusted (gamma = 1) and multiplied by the binary image that resulted from bright-field image segmentation and a second binary image that is generated by segmentation of the image using a channel-specific threshold (S3 Table).

In order to classify the pixels representing bacterial cells according to their strain, the fluorescence intensities of the RFP and YFP channels were compared. Depending on the settings, pixels were assigned to the RFP class if the RFP fluorescence intensity was greater than (>), or greater than or equal to (>=) the YFP fluorescence intensity. Pixels were assigned to the YFP class if the RFP fluorescence intensity was less than (<) the YFP fluorescence intensity.

The areas of classified segments were calculated by summation of all pixels within the classified area and stored for further processing.

References:

1. Kittler J, Illingworth J. Minimum error thresholding. Pattern Recognition. 1986; 19: 41–47.
2. Otsu N. A threshold selection method from gray-level histograms. IEEE Trans. Syst., Man, Cybern. Syst. 1979; 9: 62–66.
